# Transcriptome Analysis Identifies a 140 kb Region of Chromosome 3B Containing Genes Specific to Fusarium Head Blight Resistance in Wheat

**DOI:** 10.3390/ijms19030852

**Published:** 2018-03-14

**Authors:** Xin Li, Shengfu Zhong, Wanquan Chen, Syeda Akash Fatima, Qianglan Huang, Qing Li, Feiquan Tan, Peigao Luo

**Affiliations:** 1 Provincial Key Laboratory of Plant Breeding and Genetics, Sichuan Agricultural University, Chengdu 611130, China; sadoneli@gmail.com (X.L.); zhongsuper@163.com (S.Z.); akashfatima2247@gmail.com (S.A.F.); lovetolife@outlook.com (Q.H.); einstein355@163.com (Q.L.); tanfq@sicau.edu.cn (F.T.); 2 State Key Laboratory for Biology of Plant Diseases and Insect Pests, Institute of Plant Protection, Chinese Academy of Agricultural Sciences, Beijing 100193, China; 3 Department of Biology and Chemistry, Chongqing Industry and Trade Polytechnic Institute, Fuling District of Chongqing, Chongqing 408000, China

**Keywords:** fusarium head blight, gene island, photosynthesis, transcriptome, wheat

## Abstract

Fusarium head blight (FHB), mainly caused by *Fusarium graminearum*, is one of the most destructive fungal diseases of wheat (*Triticum aestivum* L.). Because of the quantitative nature of FHB resistance, its mechanism is poorly understood. We conducted a comparative transcriptome analysis to identify genes that are differentially expressed in FHB-resistant and FHB-susceptible wheat lines grown under field conditions for various periods after *F. graminearum* infection and determined the chromosomal distribution of the differentially expressed genes (DEGs). For each line, the expression in the spike (which exhibits symptoms in the infected plants) was compared with that in the flag leaves (which do not exhibit symptoms in the infected plants). We identified an island of 53 constitutive DEGs in a 140 kb region with high homology to the *FhbL693b* region on chromosome 3B. Of these genes, 13 were assigned to specific chloroplast-related pathways. Furthermore, one gene encoded inositol monophosphate (IMPa) and two genes encoded ribulose-1,5-bisphosphate carboxylase/oxygenase (RuBisCO). Our findings suggest that the temporary susceptibility in locally infected spikes results from the cross-talk between RuBisCO and IMPa, which blocks secondary signaling pathways mediated by salicylic acid and induces a systemic acquired resistance in the distant leaf tissue.

## 1. Introduction

Wheat (*Triticum aestivum* L.) is the most widely grown crop worldwide and accounts for approximately 20% of the calories consumed by humankind [1]. Wheat Fusarium head blight (FHB), also called “wheat cancer” or “scab”, is an economically important fungal disease that is mainly caused by *Fusarium graminearum.* FHB seriously threatens wheat production around the world [2]. The accumulation of mycotoxins produced by Fusarium, especially deoxynivalenol, in wheat and wheat products, causes acute food poisoning in humans and harms animals that consume the infected grain [3,4].

While crop management practices and chemical applications may reduce the damage caused by FHB, the development of resistant cultivars is critical for combating this disease [5,6]. The level of resistance to FHB in wheat cultivars is low, even among cultivars that are less susceptible to FHB, such as Sumai 3 and Wangshuibai. To date, no genes have been characterized that confer complete resistance to FHB in any cultivar, and this has impeded the breeding of resistant wheat [7]. The molecular mechanism underlying plant defense against *F. graminearum* infection is unknown. From both economic and human health perspectives, enhancing FHB resistance in wheat is critical for reducing yield loss and mycotoxin contamination.

FHB resistance is quantitative in nature, involving the additive effects of several genes [8]. The genetic factors underlying resistance to FHB are also highly complex, and experimental errors may have masked differences in the resistance levels among genotypes [9]. The development of resistant cultivars has been impeded because of our poor understanding of the genetic mechanisms of FHB resistance. Gene expression profile changes have provided key insights into the genetic mechanisms involved in pathogen invasion. Recently, six putative FHB resistance genes were localized within the *Fhb2* region of chromosome 6B using transcriptome analysis [10]. Thus, transcriptome analysis is a useful tool for elucidating the genetic basis of this complex trait.

In addition to causing symptoms in the spike, FHB seriously affects the developing seeds. Since the developing seeds are the most important carbohydrate sinks, and there is a dynamic balance between carbohydrate sources (e.g., the leaves) and sinks [11,12], changes in gene expression in various tissues would be expected to occur upon plant pathogen attack [13]. Therefore, it would be interesting to characterize changes in gene expression in both the leaves and the spikes of wheat plants during FHB infection and to use the expression levels in the leaves as a control when considering gene expression changes in the spike.

Genotypes with high-resistance and simple-resistance mechanisms provide a valuable pool of genes to improve crop resistance and are a prerequisite for fully understanding the resistance mechanism. In the past, studies of FHB resistance have focused on two Chinese wheat landraces, Wangshubai and Sumai 3, and their derived offspring. It is important to identify new sources of resistance against FHB in wheat, both to elucidate the resistance mechanisms and to improve cultivars.

In a previous study, we developed and identified a wheat line L693 from F_6:7_ families of a cross between wheat cultivar MY11 and the line YU25, as the donor of three disease resistance genes [14,15] that exhibit excellent resistance to stripe rust [14,16,17], powdery mildew [17], and FHB [17,18,19]. The sister line L661, derived from the same F_6:7_ families, was susceptible to stripe rust and FHB [17,18]. Further molecular tests revealed that L693 and L661 had highly similar genetic backgrounds in which 41 (2.4%) polymorphic loci were shared among 1703 loci amplified by 781 simple sequence repeat (SSR) primers [16]. This segregation in resistance showed that resistance in the F_2_ and F_2:3_ populations was inherited as two Mendelian factors, *FhbL693a* and *FhbL693b.* We then detected two major quantitative trait loci (QTLs), *Qfhs-2B* and *Qfhs-3B*, associated with FHB resistance [20]. Information regarding pedigree, inheritance, resistance response, chromosomal location, and marker diagnosis indicated that *Qfhs-3B* was different from *Fhb1*: it behaved as a single Mendelian factor and was given the gene symbol *FhbL693b* [20].

L693 is clearly an important source for disease resistance in wheat breeding programs. However, despite the successful detection and mapping of QTLs for FHB resistance in L693, the underlying molecular basis of FHB resistance remains to be elucidated.

The objective of the present study was to explore the molecular basis of FHB resistance in wheat. Two wheat genotypes, consisting of the sister lines L693 (resistant) and L661 (susceptible) were inoculated with *F. graminearum*. As the differences caused by the genetic background were minimized in these lines, we could easily identify key resistance (R) genes and the chromosomal regions responsible for FHB resistance. The expression of several randomly chosen genes in the spike (inoculated tissue; local tissue) and leaf (non-inoculated; control, distant tissue) was profiled using RNA-sequencing (RNA-Seq) and confirmed using quantitative real-time PCR (qPCR). Differentially expressed genes (DEGs) and their chromosomal distributions were compared in the two genotypes. Furthermore, we compared gene expression dynamics in the inoculated spike and non-inoculated leaf of the two genotypes, with the aim of identifying the functions and metabolic pathways of genes in the *FhbL693b* chromosomal region that are differentially expressed in the spikes and leaves of the two lines following fungal infection.

Our results provide insight into the genetic basis of FHB resistance in L693 and suggest a molecular strategy for FHB resistance based on global expression profiling. Furthermore, this work identifies potential genes for breeding applications.

## 2. Results

### 2.1. L693 and L661 Exhibit Different FHB Resistance Level, but Similar Transcriptome Sizes

**Figure 1:**
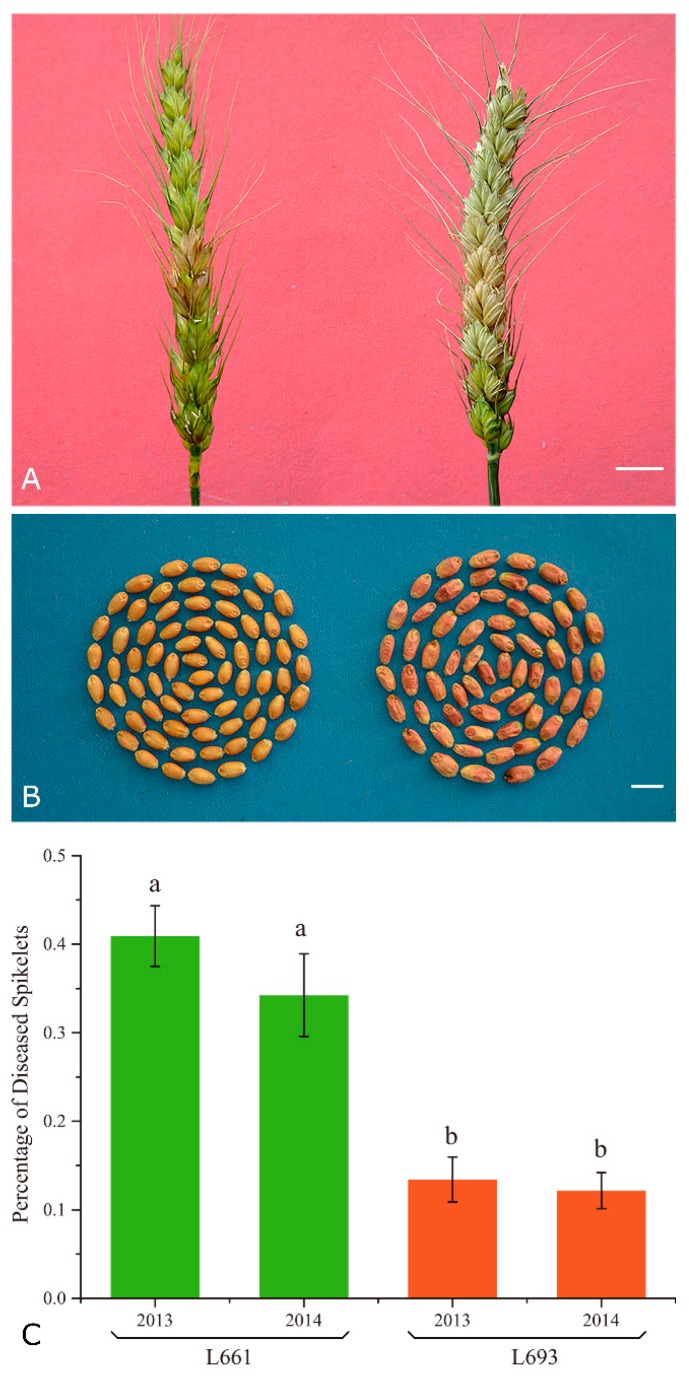
Comparisons of L693 and L661 in terms of Fusarium head blight (FHB) severity and differentially expressed genes. (**A**) Photograph of spikes of L693 (left) and L661 (right), grown under greenhouse conditions, 21 days after inoculation with *Fusarium graminearum* in 2013. For L693, disease symptoms are visible only in the inoculated spikelet, whereas, for L661, they are evident in the entire spike. Bars, 1 cm. (**B**) Comparison of kernel health between the resistant line L693 (left) and the susceptible line L661 (right) in 2013. The kernels of L693 were fully filled, while those of L661 were shriveled and pinkish. Bars, 1 cm. (**C**) Multiple comparison of the percentage of diseased spikelets (PDS) in 2013 and 2014. Error bars indicate the standard error of PDS;. Means with the same letter (above error bar) are not significantly different (*p* > 0.01) and with different letter are significantly different (*p* ≤ 0.01).

We found that L693 exhibited a high level of FHB resistance [20]; only the inoculated spikelet had dried out and died at 21 days after inoculation (DAI) with *F. graminearum*. By contrast, in L661, after the same period of time, almost the entire spike containing the inoculated spikelet had dried out and died (Figure 1A). Furthermore, the grains of L693 appeared fully filled at 21 DAI, as normal, while the grains of L661 were shriveled (Figure 1B). The percentage of diseased spikes (PDS) was significantly higher (*p* < 0.01), on the basis of multiple comparisons, in L661 than in L693 (Figure 1C).

**Figure 2:**
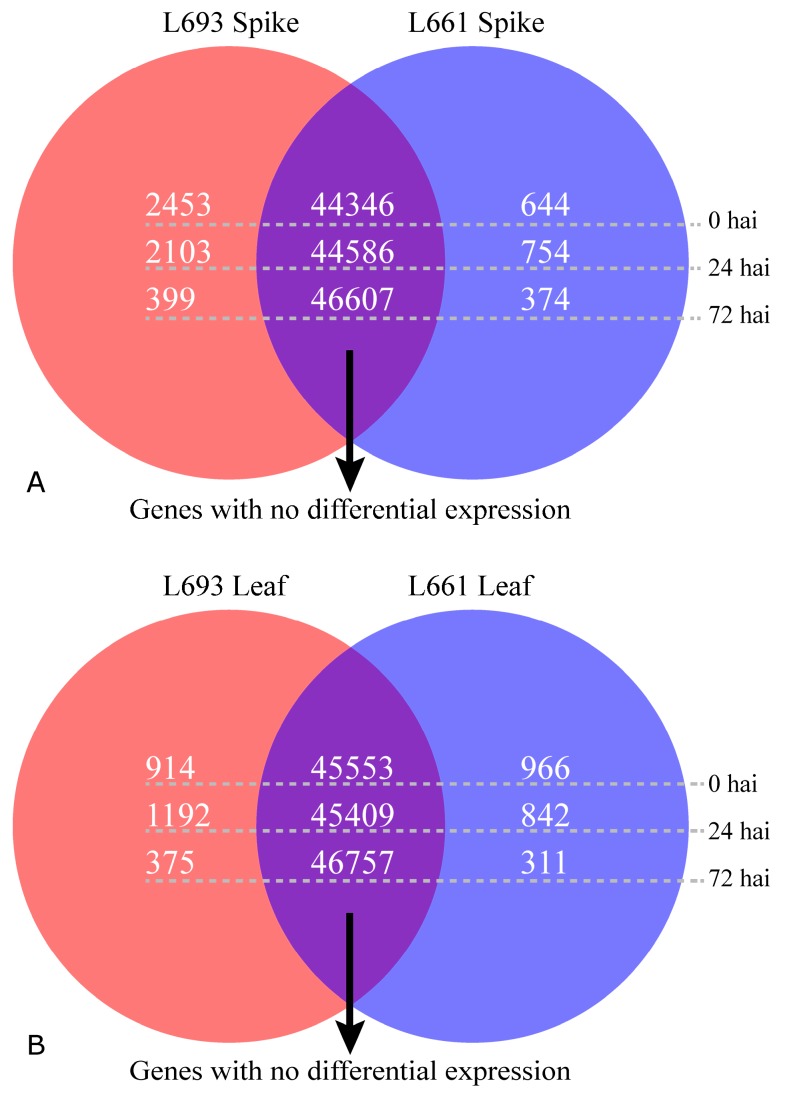
Comparison of differentially expressed genes in L693 and L661 following FHB infection. Number of upregulated genes (left, yellow circle), downregulated genes (right, blue circle), and genes without differential expression (middle, purple circle) in L693 (resistant) compared to L661 (susceptible) at 0, 24, and 72 h after inoculation (hai) in spike (**A**) and leaf (**B**).

RNA samples were taken from spike and leaf tissues of the resistant line L693 and the susceptible line L661 at three infection stages: 0, 24, and 72 h after inoculation (HAI) with *F. graminearum*, and analyzed (Table 1). Genes expressed in at least three out of 12 samples were considered expressed genes. In total, 112,484 annotated genes and 61,596 expressed genes were obtained by general filter (cutoff value ≥ 1); to get highly reliable data, a cutoff value ≥2 was applied, and, 47,443 reliable expressed genes were finally identified. Out of these 47,443 expressed genes, 2453, 2103, and 399 genes in the L693 spike were upregulated compared with the L661 spike, whereas 644, 754, and 374 genes were downregulated at 0, 24, and 72 HAI, respectively (Figure 2A). However, in leaf tissue, 914 genes were downregulated and 1192 and 375 genes were upregulated in L693, compared to 966 genes that were upregulated, and 842 and 311 that were downregulated in L661 at 0, 24, and 72 HAI (Figure 2B), respectively.

### 2.2. Validation of the Differences in Gene Expression by RT-qPCR and Clustering Analysis

**Figure 3:**
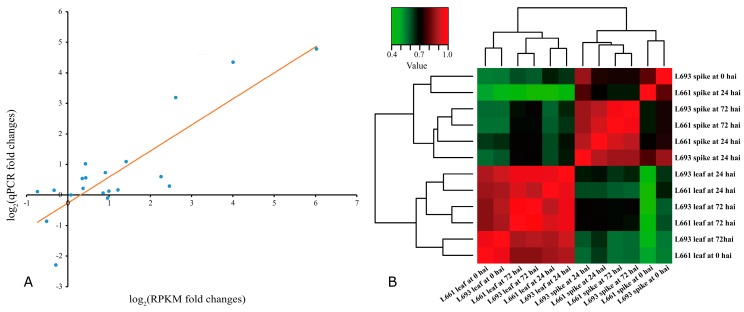
The reliability of RNA-seq data, as demonstrated by qRT-PCR (Quantitative real time polymerase chain reaction) and sample clustering. (**A**) Correlation between normalized mRNA-seq RPKM results and qRT-PCR expression values. The scatterplot shows the log_2_ fold change of RPKM and qRT-PCR expression values; a trend line is shown in red; (**B)** sample clustering based on counts of mapped Illumina reads. The dendrogram represents the hierarchical clustering of samples as determined by Euclidean distance. The heat map shows a false-color representation of the sample correlation value.

To validate the differential gene expression of the 12 samples analyzed (i.e., two genotypes, two tissues, three time points), five genes were chosen randomly for RT-qPCR analysis (Table S1). The correlation between normalized mRNA-seq RPKM (Reads Per Kilobase per Million mapped reads) results and qRT-PCR expression values were high (*R*^2^ = 0.73) (Figure 3A, Supplementary Material 2: Table S1). In addition, 12 clustering samples based on Illumina read counts mapped against the Ensembl wheat genome sequence showed that the tissue type had the biggest effect on the changes in gene expression, followed by the time after inoculation and the genotype of wheat used (Figure 3B). The clustering results showed that the difference in gene expression between the spike and leaf was greater than that between the genotypes.

### 2.3. Differential Expression Analysis Revealed Tissue-Specific Expression Tendencies

**Figure 4:**
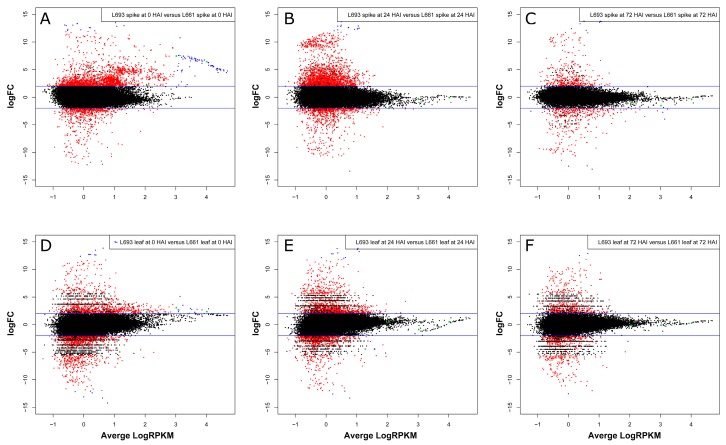
Log-fold change (logFC) against average logRPKM in different genotypes. (**A**–**F**) Differentially expressed genes with a false discovery rate (FDR) of less than 5% and a log-fold change larger than 2 are highlighted in red. In each panel, the red dots above the upper blue line (logFC > +2) represent the upregulated genes in L693 or the downregulated genes in L661; the red dots blow the lower blue line (logFC < −2) represent the upregulated genes in L661 or the downregulated genes in L693. The blue dots represent gene(s) with extreme values of −12 > logFC > 12 or with an average logRPKM > 3. The green symbols represent genes of interest in this study; rectangle for MSTRG.24516, circle for MSTRG.24551, triangle for MSTRG.24552.

Using the edgeR software [21], more DEGs were identified in the spike than in the leaf of both L693 and L661 plants. Among the 3097 DEGs identified in the spike at 0 HAI, 14 were strongly upregulated (i.e., had logFC values greater than +12), but only two were strongly downregulated (i.e., with logFC values lower than −12). High expression abundance (i.e., average logRPKM values greater than 3) was found in 84 of the 2453 upregulated genes (shown in blue in Figure 4A) compared to the 644 downregulated genes. At 24 HAI, there were 2857 DEGs in the spike, including 13 that were strongly upregulated and only one that was strongly downregulated in L693 compared to L661 (Figure 2A and Figure 4B, Supplementary Material 2: Table S2). At 72 HAI, 773 DEGs were observed in the spikes of the two genotypes, including seven that were strongly upregulated and three that were strongly downregulated in L693 compared to L661 (Figure 2A and Figure 4C, Supplementary Material 2: Table S2). Seven of the 374 downregulated genes had higher average logRPKM values than the 399 upregulated genes.

In leaf tissues, there were 1880 DEGs at 0 HAI between the two genotypes; these included 11 strongly upregulated genes and six strongly downregulated genes in L693 compared to L661. Fifteen of the 914 upregulated genes had high expression abundance compared to the 966 downregulated genes. At 24 HAI, there were 2034 DEGs in the two genotypes, including 10 that were strongly upregulated and three that were strongly downregulated in L693 compared to L661. At 72 HAI, 686 DEGs were detected in the two genotypes, only two of which were strongly upregulated and one of which was strongly downregulated (Figure 2B and Figure 4F, Supplementary Material 2: Table S2).

We identified some similar changes in gene expression between the spike and leaf tissues (Figure S1). The number of upregulated genes in the spike compared to the corresponding leaf was greater than that of the downregulated genes at each time point in both L693 and L661 plants. The change in gene expression in the L661 spike from 0 to 24 HAI was greater than that of L693 (Supplementary Material 1: Figure S2d,j). By contrast, there was a greater change in gene expression in the L693 spike compared to L661, both from 0 to 72 HAI and from 24 to 72 HAI (Supplementary Material 1: Figure S2e,f,k,l). Many of the upregulated genes in the L661 spike from 0 to 24 HAI and from 0 to 72 HAI had larger logRPKM values than the corresponding downregulated genes (Supplementary Material 1: Figure S2d,e). A greater number of DEGs were seen in the L693 spike from 24 to 72 HAI than in the L661 spike (Supplementary Material 1: Figure S2f,l). Moreover, in L693, there were more downregulated genes than upregulated genes, while in L661 there were fewer downregulated genes than upregulated genes (Supplementary Material 2: Table S3). The expression of genes in the leaves of both genotypes were similar; the largest difference between L693 and L661 spikes occurred from 0 to 24 HAI, while that in leaves was from 24 to 72 HAI (Supplementary Material 1: Figure S2).

### 2.4. DEGs Have a Biased Chromosomal Distribution

**Figure 5:**
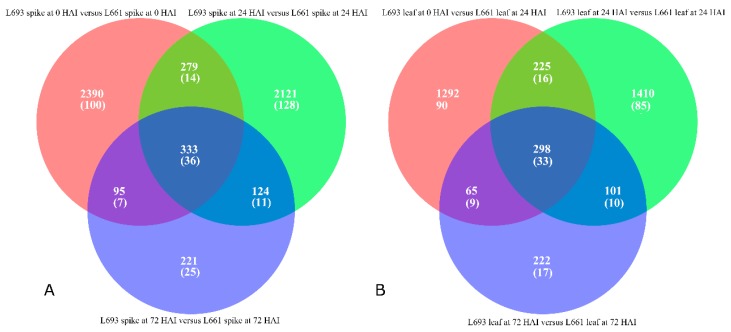
Venn diagram showing the number of differentially expressed genes shared by L693 and L661 at 0 h (red), 24 h (green), and 72 h (blue) after inoculation for the spike (**A**) and leaf (**B**). The numbers without parentheses represent the differentially expressed genes across the whole genome; the numbers in parentheses represent the differentially expressed genes on wheat chromosome 3B.

The chromosomal distribution of DEGs was biased (Figure 5). In the spike, 36 (10.8%) of 333 genes differentially co-expressed in L693 and L661 at the three time points examined were mapped to chromosome 3B. In the leaf, 33 (11.1%) of 298 genes differentially co-expressed in L693 and L661 at the three time points were mapped to chromosome 3B. In addition, there was a similar number of total DEGs and DEGs on chromosome 3B between L693 and L661 at the three time points (Figure 5, Supplementary Material 2: Table S4). The genes that were differentially co-expressed in the spike and leaf at three points in both L693 and L661 exhibited no biased distribution on chromosome 3B (Supplementary Material 1: Figure S3). In both L693 and L661 spikes, no biased distribution of co-expressed DEGs (24 vs. 0, 72 vs. 0 and 72 vs. 24) on chromosome 3B was found, but the number of co-expressed DEGs (24 vs. 0, 72 vs. 0 and 72 vs. 24) mapping to chromosome 3B in the leaves of L693 and L661 plants at the three time points was only 9 (2.8%) and 15 (3%) out of 317 and 496, respectively, which is fewer than the chromosomal average value of about 5.2% (Supplementary Material 1: Figure S4, Supplementary Material 2: Table S4).

### 2.5. A 140 kb Differential Expression Island Exists on Chromosome 3B

**Figure 6:**
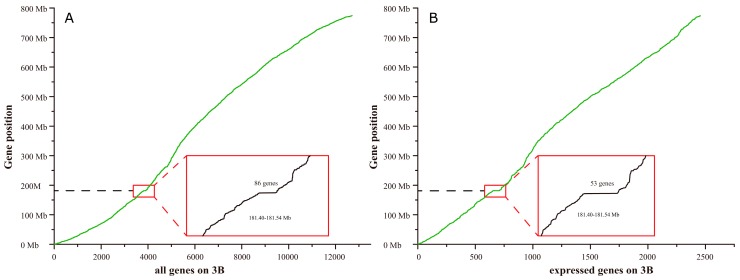
Distribution of genes on wheat chromosome 3B. (**A**) Eighty-six genes annotated by the Genetics Diversity Ecophysiology of Cereals (GDEC) group at the French National Institute of Agronomic Research (INRA) located in region 181.40–181.54 Mb of wheat chromosome 3B. (**B**) Fifty-three expressed genes located in region 181.40–181.54 of chromosome 3B in this study. The insets show enlargements of the boxed regions.

We then searched sequence data (Available online: http://plants.ensembl.org/Triticum_aestivum) for genes on wheat chromosome 3B. We identified 8571 annotated genes with a total length of 774,434,471 bp, excluding scaffolds, and an average length of 90.4 kb. Further analysis of the reference sequence revealed a gene island in the region between 181.40 and 181.54 Mb; this 140 kb sequence contained 73 annotated genes with an average density of 1.9 kb. Reliable transcription products of 1350 (16.8%) out of 8571 annotated genes were detected with an average density of 573.7 kb, while 42 (57.5%) out of 73 annotated genes in the 3B chromosomal region from 181.40 to 181.54 Mb were transcribed. RNA-Seq data led to the identification of 4136 unannotated transcribed genes that may encode unknown RNAs or proteins; these genes are hereafter referred to as novel transcribed genes (NTGs). A total of 12,707 annotated genes (including previously annotated genes and NTGs) were identified on chromosome 3B, with an average gene density of 60.9 kb, while 86 genes were found on chromosome 3B from 181.40 to 181.54 Mb, with an average gene density of 1.6 kb (Figure 6). Furthermore, 2456 (19.3%) out of 12,707 genes on chromosome 3B exhibited reliable expression with an average gene density of 315.3 kb, while 53 (61.6%) out of 86 genes were reliably expressed on chromosome 3B from 181.40 to 181.54 Mb, with an average gene density of 2.6 kb.

### 2.6. Genes in the 140 kb Expression Island Exhibit Higher Constitutive Expression in the FHB Resistance Genotype

**Figure 7:**
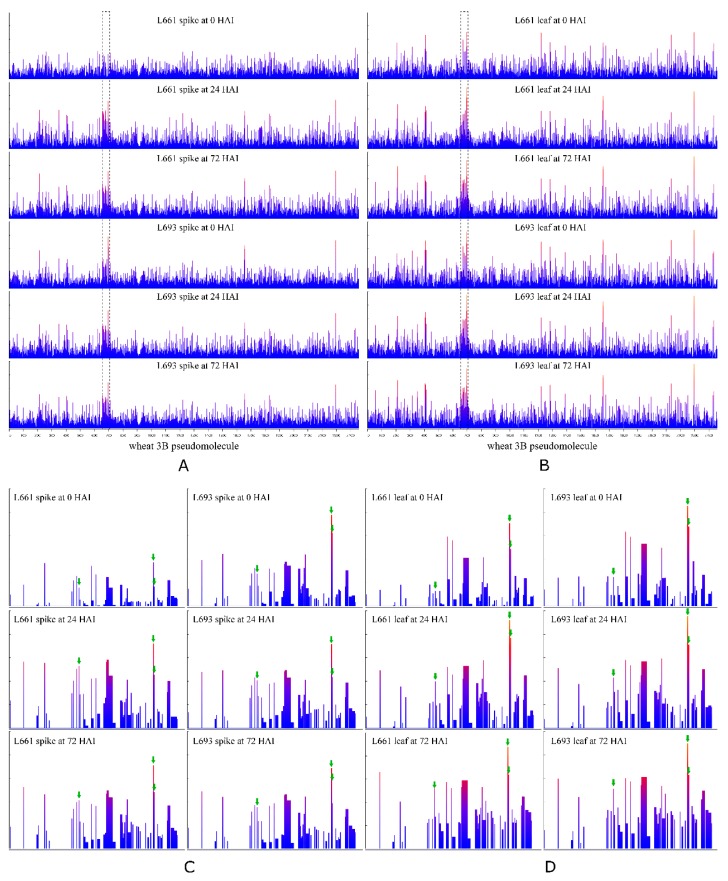
Distribution of gene expression levels on chromosome 3B in all 12 samples. The *x*-axis represents the chromosome range 0–774.43 Mb. The *y*-axis represents the normalized log (RPKM+1) range 0–1. The region included in the dashed rectangle indicates the 181.40–181.54 Mb region. Distribution of gene expression level on chromosome 3B for six spike samples (**A**) and six leaf samples (**B**). Distribution of gene expression level in the gene island at region 181.40–181.54 Mb of chromosome 3B for six spike samples (**C**) and six leaf samples (**D**).

Twenty-seven out of 53 genes had a higher expression level (i.e., −2 < logFC < 2) in the L693 spike than in the L661 spike at 0 HAI, while no difference in gene expression was observed (i.e., −2 < logFC < 2) between the spikes of L693 and L661 at 24 or 72 HAI. In the leaf, 9, 2, and 1 out of the 53 genes were differentially expressed in L693 and L661 at 0, 24, and 72 h, respectively. For all differentially expressed genes, the expression level in L693 was visibly higher than that in L661. Furthermore, in both the spike and leaf, all 36 genes expressed in L693 and L661 at 0 HAI were upregulated in L661 from 0 to 24 HAI, while only 24 genes were upregulated in L693 during that period (Figure 7, Supplementary Material 2: Table S5).

### 2.7. DEGs in the 140 kb Expression Island Are Mainly Involved in Chloroplast Function

Of the 53 DEGs identified, 42 were previously annotated and 11 (with transcript ID name “MSTRG” in Table S5) were newly annotated in this study. Using the BLAST program, DEGs were identified that had high levels of nucleotide sequence similarity with annotated genes for chloroplast-related pathways (39; 73.6%), mitochondrion-related pathways (3; 5.7%), and genes encoding putative proteins without known functions (7; 13.2%). Four (7.5%) DEGs showed no similarity to any previously identified genes (Table S5). Further Kyoto Encyclopedia of Genes and Genomes (KEGG) pathway enrichment analysis revealed that 13 (24.5%) of the 53 DEGs were assigned to a specific pathway (Supplementary Material 2: Table S5); of these, 7 (53.8%) were involved in oxidative phosphorylation pathways (Table 2). Some DEGs were involved in other known pathways, including purine metabolism, glyoxylate and dicarboxylate metabolism, biosynthesis of antibiotics, carbon fixation in photosynthetic organisms, thiamine metabolism, pyrimidine metabolism, galactose metabolism, phenylpropanoid biosynthesis, and starch and sucrose metabolism (Supplementary Material 2: Table S5). Thirteen DEGs showed wide pleiotropism; for example, *MSTRG.24512*, which encodes ec:3.6.1.3-adenylpyrophosphatase and ec:3.6.1.15-phosphatase, participates in four pathways including purine metabolism, thiamine metabolism, galactose metabolism, and starch and sucrose metabolism (Supplementary Material 2: Table S5). Eleven (84.6%) of these 13 DEGs are involved in chloroplast-related metabolism, and 2 of these DEGs are involved in mitochondrion-related metabolism. Further analysis found that 9 and 4 of the 13 DEGs were differentially expressed between the two genotypes at 0 DAI in the spike and leaf, respectively, with line L693 exhibiting higher constitutive expression than line L661. We paid specific attention to three interesting constitutively expressed genes that were induced upon inoculation with *F. graminearum*: one gene encoding inositol monophosphate (IMPa) and two genes encoding ribulose-1,5-bisphosphate carboxylase/oxygenase (RuBisCO).

## 3. Discussion

In this study, we used RNA-Seq to characterize genes that were differentially expressed in the resistant the L693 genotype, which carries *FhbL693b,* and the susceptible L661 genotype, which lacks *FhbL693b*, following *F. graminearum* infection and compared the expression of genes in the spikes and leaves of both lines at various time points. Our findings pave the way for developing an integrated model for explaining the response to *F. graminearum* infection in wheat.

### 3.1. The Reliability of Original Data Was Important for Comparing Transcriptome Analyses

We collected RNA-Seq samples at three time points (0, 24, and 72 hai) from two genotypes (L693 and L661) and two tissues (spike and leaf), with the aim of analyzing the effect of genotype, tissue type, and time on gene expression changes. No mock inoculation was performed, as this would not have provided any additional information in the complex environment of the field, but would have increased the cost of the sequencing. As gene expression analysis is considered to be equally accurate in the field and the greenhouse [22], we performed all the inoculations and sample harvests under field conditions.

The difference in the PDS was significant between the two genotypes but was not affected by the experimental conditions used (i.e., 2013 vs. 2014; field vs. greenhouse) (Figure 1C), which indicated that infection in the field was as effective as in the greenhouse. After infection, the change in gene expression exhibited a similar tendency and degree between the L693 and L661 lines (Figure S1, Supplementary Material 1: Figure S2). Further analysis of 12 samples showed that the greatest difference in gene expression following *F. graminearum* infection occurred between different tissues (spike vs. leaf), while the smallest difference was observed between the resistant and susceptible genotypes (Figure 3B). This could be due to the fact that L693 and L661 have similar genetic backgrounds [16,20,23]. The fact that the greatest difference in gene expression occurred between the spike and leaf indicated that the transcriptome data were reliable to some degree, which could be a real response after *F. graminearum* infection. To further validate the data, we randomly selected five genes for RT-qPCR analysis (Supplementary Material 2: Table S1). A high correlation (*R*^2^ = 0.73) between RPKM and RT-qPCR expression values (Figure 3, Supplementary Material 2: Table S1) indicated that the RNA-seq data were indeed reliable.

### 3.2. DEGs in Both the Leaf and Spike Play Key Roles in FHB Resistance Establishment

Following pathogen attack, plants must redistribute energy and resources from the primary metabolism to the induced reaction [24]. Previous studies of wheat spike diseases such as FHB have usually focused on gene expression changes only in local spike tissue, whereas studies of wheat leaf diseases, such as stripe rust and powdery mildew, have focused on gene expression changes only in the local leaf tissue [13,23]. Gene expression changes in distant tissues of plants infected by pathogens, such as the leaves of wheat plants infected by *F. graminearum,* have not been documented.

In the present study, a comparison of the L693 and L661 lines showed that more genes were upregulated than downregulated in the spike after *F. graminearum* infection, while a similar number of genes were expressed in the leaves of these lines at 0 h (Figure 2a). Interestingly, the spike exhibited fewer upregulated genes than downregulated genes, while the number of genes expressed in the leaf increased at 24 h. At 72 h, the number of upregulated genes was almost equal to that of the downregulated genes in both the spike and the leaf (Figure 2A and Figure 4). Many genes that were constitutively expressed at higher levels in the spike were induced in the leaves of the FHB-resistant genotype, but not in those of the FHB-susceptible genotype. Though the pore-forming toxin-like (PFT) gene at *Fhb1* was also constitutively expressed in the spike of the FHB-resistant genotype, the expression in uninoculated leaves was not determined [25]. As FHB resistance mainly results from the systemic acquired resistance induced by infection of the pathogen, we propose that the strongly induced gene expression observed in the leaf may play an important role in FHB resistance.

### 3.3. The Gene Island and 140 kb Differential Gene Expression Island on Chromosome 3B Were Highly Associated with FHB Resistance

A gene island is a gene-rich genomic region [26]. Previous studies demonstrated that gene islands are abundant in the wheat genome [27,28,29]. In this study, we firstly found that genes that were differentially expressed following *F. graminearum* infection exhibited an obvious bias in chromosomal location to chromosome 3B (Figure 5 and Figure 6, Table 2). In both the spike and the leaf, the differentially co-expressed genes on chromosome 3B in the L693 and L661 lines represented more than 10% of the total number of differentially co-expressed genes at the three time points (Table 2). Furthermore, in both L693 and L661 leaves, the differential co-expressed genes on chromosome 3B represented 3% or less than the total number of differentially co-expressed genes at each time point (Supplementary Material 1: Figure S3, Table S2), indicating that the differential gene expression on chromosome 3B was highly related to FHB resistance. Secondly, a gene island from 181.40 to 181.54 Mb was identified. The average gene length within the gene island was 1.6 kb, whereas it was 60.9 kb on the whole of chromosome 3B. Thirdly, the region from 181.40 to 181.54 Mb of chromosome 3B was also a gene expression island, in which a proportion (61.6%) of the expressed genes was markedly higher than that (19.3%) on the whole of chromosome 3B. Thus, a gene island and a gene expression island exist in region 181.40 to 181.54 Mb of chromosome 3B.

*FhbL693b* was different from the well-known FHB resistance QTL *Fhb1* on 3BS, on the basis of genetic mapping results [20]. The chromosomal region from 181.40 to 181.54 Mb was also different from the *Fhb1* region as determined by physical mapping [25]. In addition, the gene island from the wheat reference sequence and the gene expression island from the transcriptome were located within the FHB resistance QTL *FhbL693b*. Together, these findings indicate that the gene island and the gene expression island in the chromosomal region from 181.40 to 181.54 Mb are functional FHB resistance gene islands, which could mediate FHB resistance both via genomic constitutive expression and induced expression after infection by *F. graminearum*.

### 3.4. Constitutive Differential Expression of Genes in the 181.40 to 181.54 Mb Region of Chromosome 3B Plays a Key Role in FHB Resistance

Many genes in the chromosomal region from 181.40 to 181.54 Mb exhibited markedly higher constitutive expression in both the spike and the flag leaf of L693 as compared to L661 (Supplementary Material 2: Table S5). We found that 27 and 9 DEGs exhibited markedly higher constitutive expression in the spike and flag leaf, respectively, of L693 plants than in those of L661 plants (Supplementary Material 2: Table S5). Interestingly, all 53 DEGs were upregulated in the spike of L661 at 24 HAI as compared to 0 HAI, and 41 of these were markedly upregulated. However, in the spike of L693 plants, only 12 of the 39 upregulated genes were markedly upregulated, and 1 gene exhibited downregulated expression (Supplementary Material 2: Table S5). These observations indicated that the higher constitutive expression of these genes in the region 181.40 to 181.54 Mb of chromosome 3B, especially in the spike, might play a key role in FHB resistance. A recent publication reported the pore-forming toxin-like (PFT) gene as a candidate gene of *Fhb1* exhibiting a higher constitutive expression in R-NIL, resulting in a sharp decline in transcript levels after the emergence of spikes [25], which further supports our proposal that increased constitutive expression plays a vital role in FHB resistance.

### 3.5. Photosynthesis Is Possibly Involved in FHB Resistance 

The chloroplast is involved in energy production, redox homeostasis, and retrograde signaling, and these processes collectively participate in the plant immune response [30]. The plant’s response to pathogen attack is closely linked to a change in energy metabolic pathways such as the photosynthesis [31]; therefore, the process could be tracked and quantified using photosynthesis-related parameters, including net photosynthetic rate and chlorophyll fluorescence [19,32,33]. Historically, immunity and photosynthesis were studied separately. Therefore, discussing the cross-talk between photosynthesis and immunity would be useful for plant protection. In the present study, we found that 13 (24.5%) out of 53 DEGs were assigned to a specific pathway and 7 (53.8%) out of 13 genes in region 181.40 to 181.54 Mb of chromosome 3B were involved in oxidative phosphorylation pathways (Supplementary Material 2: Table S5). We also found that specific metabolic pathways of 11 genes occur in the chloroplast and only two, encoding the apocytochrome b genes MSTRG.24508 and MSTRG.24508, occur in the mitochondrion (Supplementary Material 2: Table S5), indicating that photosynthesis in both the spike and the leaf could play a vital role in regulating wheat resistance to FHB.

By conducting a comparison of differential constitutive gene expression and induced changes in expression following *F. graminearum* infection in two genotypes, we further identified three intriguing genes: MSTRG.24516 (encoding inositol monophosphatase (IMPa)) and MSTRG.24551 and MSTRG.24552 (encoding ribulose-1,5-bisposphate carboxylase/oxygenase, RuBisCO). IMP biosynthesis catalyzed by IMPa is crucial in multicellular eukaryotes, while IMPa is required for the breakdown of inositol (1,4,5)-trisphosphate, an important secondary messenger involved in Ca^2+^ signaling [34,35]. A recent study demonstrated that IMP inhibited the salicylic acid-dependent pathogen defense responses triggered by reactive oxygen species (ROS) [36]. This information supports our hypothesis that the differential expression of MSTRG24516 is involved in wheat FHB resistance.

In addition, RuBisCO acts as a key regulator that controls the balance between photosynthesis and photorespiration. An oxidative burst mainly resulting from an increase in photorespiration during primary fungal pathogen infection produces various ROS [37], which negatively influences resistance or promotes disease development [38]. *F. graminearum* infection can be divided into biotrophic and necrotrophic stages, and therefore, it uses both living and dead tissues for nutritional purposes, which causes the host to be less resistant to FHB than to other wheat diseases. Tissue necrosis caused by the primary ROS during pathogen infection increases wheat host susceptibility to the necrotrophic form of *F. graminearum*, but resistance to its biotrophic form. On the one hand, pathogen-induced ROS are signaling molecules that trigger systemic resistance. This type of resistance is characterized by the rapid generation of hydrogen peroxide (H_2_O_2_), which acts as a secondary messenger that induces the expression of defense genes [39]. On the other hand, ROS could directly act as a weapon to resist pathogens, because these molecules are also harmful to pathogens [40]. In this study, there was a markedly higher constitutive expression of MSTRG.24551 and MSTRG.24552 in both the spike and the leaf of L693 as compared to L661, indicating that MSTRG.24551 and MSTRG.24552, which encode RuBisCO, are also involved in wheat FHB resistance

To further elucidate the cross-talk between IMP and RuBisCO, it is useful to understand their mechanisms of action in the secondary signaling pathway. In the spikes of L693, but not L661, plants, we observed a sharp decrease in RuBisCO expression following local infection (Supplementary Material 2: Table S5), and this decrease was accompanied by an increase in the net photosynthetic rate (Pn) (Supplementary Material 1: Figure S5), indicating that photorespiration could be blocked to some degree, resulting in less oxidative stress. Less oxidative stress not only has a positive effect on IMP biosynthesis, but also possibly blocks the plant resistance signaling pathway mediated by SA. Interaction between IMP and RuBisCO may lead to temporary susceptibility and short-lived resistance in the locally infected spikes of L693 and L661 plants, respectively, following primary physiological signaling that triggers the secondary signaling pathway in distant tissue such as the leaf. In the distant uninfected leaves of L693 plants, the increased RuBisCO (Supplementary Material 2: Table S5), as compared to L661, was used for photorespiration, as there was a decrease in *P_n_* (Supplementary Material 1: Figure S5a), causing high oxidative stress, which contributed to the secondary signaling pathway mediated by SA (Salicylic acid), and this produced systemic acquired resistance against wheat FHB. Comparisons of SOD (Superoxide dismutase) and CAT (Catalase) contents in the two lines over time (Supplementary Material 1: Figure S5b,c) would support our assertion. Additionally, the chloroplast protein NRIP1, which is responsible for Tobacco mosaic virus recognition [41], the Arabidopsis protein PHYTOPHTHORA1, which imparts resistance to *Phytophthora brassicae* [42], and the wheat kinase START1 resistance protein (WKS1) [43] are localized in the chloroplast, supporting the view that RuBisCO is also involved in FHB resistance.

**Figure 8:**
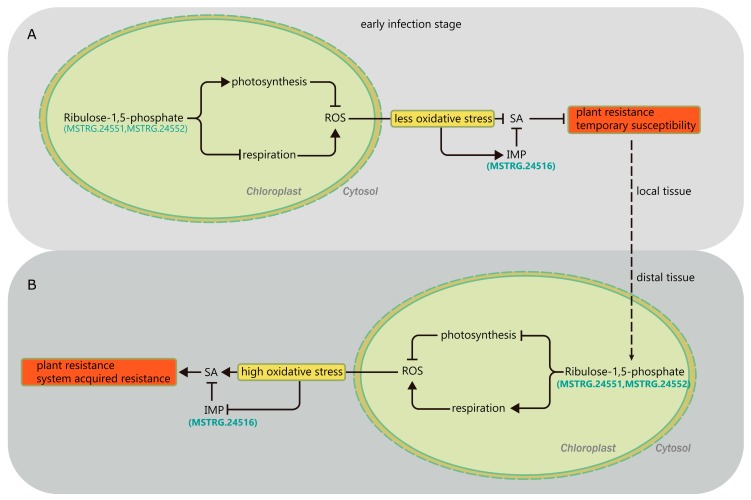
A proposed working model of the roles of MSTRG.24516, MSTRG.24551, and MSTRG.24552 in the regulation of FHB resistance in wheat line L693. (**A**) In the early infection stage, FHB induces temporary susceptibility locally in a tissue; (**B**) this then leads to system-acquired resistance in distal tissues.

In conclusion, changes in the balance between photosynthesis and photorespiration regulated by RuBisCO could lead to low ROS and/or high IMP levels (Figure 8), both of which would block secondary signaling pathways mediated by SA, causing a brief susceptibility in the local spike of FHB-resistant L693. Subsequently, the short susceptible response would induce distant leaf tissue to produce systemic acquired resistance through the opposite regulation of the above pathways mediated by SA.

## 4. Materials and Methods

### 4.1. Plant and Pathogen Materials

FHB-resistant wheat line L693 and FHB-susceptible line L661 were selected and developed from F_6:7_ families derived from a cross between the FHB-susceptible wheat cultivar MY11 and the FHB-resistant line YU25 [17,18,20]. Several years of continuous resistance testing confirmed that L693 was strongly, stably, and consistently resistant to FHB [17,18]. Further studies showed that FHB resistance in all tested populations was inherited as two Mendelian factors, *FhblL693a* and *FhbL693b* [20]; therefore, this line was selected for further study of the FHB resistance mechanism. The monosporic isolate of *F. graminearum* Fg 4 was used for fungal infection; this was kindly provided by Professor Ma Zhengqiang (Nanjing Agricultural University, Nanjing, China).

### 4.2. F. graminearum Spore Production and Inoculation

A macroconidial suspension was produced and harvested by a method previously described [19]. Wheat spikes from L693 and L661 were point-inoculated by injection with a freshly prepared spore suspension at the anthesis stage, and four florets of two central spikelets per spike were used. Each floret was injected with 5 µL of macroconidial suspension (200 macroconidia per µL) and was covered by a plastic bag to maintain humidity; the bags were removed 72 h after inoculation. The whole spikes and flag leaves of the two genotypes were harvested at 0 h (inoculated with fresh water), 24 h, and 72 h.

### 4.3. Experimental Design and Sample Harvesting

To understand the mechanism of FHB resistance under natural field conditions, seeds of L693 and L661 were grown in fields at the Wenjiang Experimental Station of Sichuan Agricultural University (latitude 30°43′ N, longitude 103°52′ N), in southwest China during the 2012–2013 wheat growing season, which was temperate and rainy (annual average temperature of 17 °C and rainfall of 1350–1580 mm). The field experiments used a randomized complete block design with three replications. Each block consisted of twelve 3 m-long rows (with a row spacing of 33 cm), with an interplant width of 15 cm. Plants randomly chosen from each population, which were distributed across three blocks, were marked at spike emergence (about 21 March 2013) for future inoculation. Previously described methods were used to control leaf diseases such as stripe rust and powdery mildew, and for fertilizer and pest management [12]. The inoculation of the samples was started at 9 a.m. To detect gene expression changes in the inoculated local spike, the gene expression levels were compared with those in a distal non-inoculated leaf. The 0 h samples (non-inoculated) were used as a control. Therefore, 12 samples (two genotypes, two tissues, and three time points) were prepared for RNA extraction per block. FHB resistance was also evaluated in plants grown in greenhouses at Kansas State University (Manhattan, KS 66506, USA) according to a previously described program [17].

### 4.4. RNA Extraction and RNA-Sequencing

Total RNA was isolated using TRIzol reagent (Invitrogen, Carlsbad, CA, USA) according to the manufacturer’s protocol. The total RNA of three leaves and three spikes of the same wheat line from each block at the same time point were mixed in equal amounts for transcriptome sequencing. A transcriptome library with fragments ranging in size from 200 to 700 bp was prepared using an Illumina Kit and sequenced on an Illumina HiSeq™ 2000 (Illumina, Inc. 9885 Towne Centre Drive, San Diego, CA, USA), using paired-end technology in a single run. Single-end sequencing ensured that the sequencing depth of each sample exceeded 5 Gb. RNA-Seq was executed by ABlife Inc. (Wuhan, China).

### 4.5. RNA-Seq Data Quality Control and Alignment Statistics

Raw reads were trimmed if they contained more than 2-N bases and then processed by adaptor clipping and removal of low-quality bases. Short reads of less than 16 nt in length were also removed. FASTX-Toolkit (v 0.0.13) (Gordon, Cold Spring Harbor, NY, USA) was used to obtain clean reads, and Fastqc (Available online: http://www.bioinformatics.babraham.ac.uk/projects/fastqc/) was used to check the quality of the clean reads. After that, Tophat2 (The Center for Computational Biology at Johns Hopkins University, Baltimore, MD, USA) [44] was used to align the clean reads to the wheat reference genome assembly version 30 based on IWGSC wheat genome refrence [45] from Ensembl Plants [46], the parameters of tophat2 were set to end-to-end method with two mismatches allowed. Then, stringtie [47] was used to evaluate the abundance of each gene in 12 samples. Genes with a total abundance lower than 24 alignments in all 12 samples (average cutoff value ≥2) and expressed less in 3 samples were filtered. Then edgeR [21] were used to calculate the RPKM value (RPKM represents reads per kilobase and per million) according to the abundance data for each gene in the 12 samples. R package Sushi [48] was used to explore the gene expression distribution on chromosome physical maps.

### 4.6. Reliable Analysis of Sequencing Data and Quantitative Real-Time PCR

To further demonstrate the reliability of the RNA-Seq data, five genes were chosen randomly from the expressed genes for quantitative qRT-PCR experiments. The details of the primer sequences and descriptions of the genes are shown in (Supplementary Material 2: Table S1). Complementary DNA (cDNA) for real-time quantitative reverse transcription PCR (qRT-PCR) was synthesized by M-MLV (Invitrogen). Each 20 µL reaction contained 4 µg RNA, 2 µL cDNA diluted with pure water (1:20), and 9 µL RealMasterMix (SYBR solution; TIGEN). The MiniOpticon Time PCR Detection System was used for qPCR, as previously described [23]. Three biology replicates for each sample were tested, and the relative expression level was calculated by the 2^−ΔΔCT^ method [49]. Then, correlations between the normalized RPKM results and the qRT-PCR expression values were performed to determine the reliability of RNA-seq data.

### 4.7. Differential Gene Expression Analysis and Identification of Hotspot Expression Polymorphism

Differential gene expression between the spike and leaf of L693 or L661 at various time points was measured using edgeR [21] software. For each gene, the *p* value was computed, and the significance threshold to control FDR (false discovery rate) at a given value was calculated. The fold changes were also estimated with the edgeR statistical package. Genes with FDR < 5% and log-fold change >2 were considered as DEGs.

### 4.8. Bioinformatic Analysis of Pathway-Specific Gene Function in the FhbL693b Region

Identified unigenes were aligned to the NR database to get annotations using BLASTX and BLASTN. Blast2go [50] was used to get the GO annotation of genes. Putative physiological and functional categories were assigned as per the GoFigure Gene Ontology (GO) annotation [12].

## Figures and Tables

**Table 1:** RNA-seq read information.

Tissue	Samples	Raw Data	Clean Reads	Mapped	Mapped %	Multiple	Multiple %	Unique	Unique %
spike	L661 spike at 0 HAI	48,619,708	44,064,636	31,413,442	71.3%	7,948,442	25.3%	23,472,782	74.7%
	L693 spike at 0 HAI	54,638,926	50,729,857	37,433,618	73.8%	15,223,441	40.7%	22,210,177	59.3%
	L661 spike at 24 HAI	80,401,044	14,261,236	6,799,856	47.7%	2,277,018	33.5%	4,522,838	66.5%
	L693 spike at 24 HAI	56,001,617	37,706,168	24,971,426	66.2%	7,770,208	31.1%	17,201,218	68.9%
	L661 spike at 72 HAI	64,589,048	47,444,396	29,577,848	62.3%	9,209,005	31.1%	20,368,843	68.9%
	L693 spike at 72 HAI	73,276,052	52,378,107	33,229,807	63.4%	9,061,381	27.3%	24,168,426	72.7%
leaf	L661 leaf at 0 HAI	74,642,980	40,067,567	28,798,631	71.9%	9,266,948	32.2%	19,983,610	69.4%
	L693 leaf at 0 HAI	53,896,907	37,849,318	18,909,880	50.0%	8,498,338	44.9%	10,411,542	55.1%
	L661 leaf at 24 HAI	52,460,707	45,960,283	35,981,574	78.3%	20,792,164	57.8%	15,189,410	42.2%
	L693 leaf at 24 HAI	54,132,118	45,690,464	32,199,399	70.5%	20,182,030	62.7%	12,017,369	37.3%
	L661 leaf at 72 HAI	50,556,369	26,746,381	18,315,598	68.5%	9,430,647	51.5%	6,319,347	34.5%
	L693 leaf at 72 HAI	49,560,263	26,728,939	18,240,700	68.2%	10,006,376	54.9%	8,234,324	45.1%

HAI: Hours after inoculation.

**Table 2:** Pathways associated with the 13 genes in the gene island at region 181.40–181.54 Mb of wheat chromosome 3B.

Pathways	Enzyme	Definition	Seq Nu.	Sequence
Oxidative phosphorylation	ec:1.10.2.2—reductase	ubiquinol-cytochrome c reductase cytochrome b/c1 subunit	7	MSTRG.24508
	ec:1.6.99.3—dehydrogenase	NADH dehydrogenase		MSTRG.24509, MSTRG.24524, MSTRG.24557, MSTRG.24558, MSTRG.24527, TRAES3BF007300290CFD_g
	ec:1.9.3.1—oxidase	cytochrome c oxidase cbb3-type subunit I		MSTRG.24509
	ec:1.6.5.3—reductase (H^+^-translocating)	NADH:ubiquinone reductase (H+-translocating)		MSTRG.24509, MSTRG.24524, MSTRG.24557, MSTRG.24558, MSTRG.24527, TRAES3BF007300290CFD_g
Purine metabolism	ec:3.6.1.3—adenylpyrophosphatase	adenosinetriphosphatase	3	MSTRG.24512, MSTRG.24554
	ec:3.6.1.15—phosphatase	nucleoside-triphosphatase		MSTRG.24512, MSTRG.24554
	ec:2.7.7.6—RNA polymerase	DNA-directed RNA polymerase subunit alpha		MSTRG.24544
Glyoxylate and dicarboxylate metabolism	ec:4.1.1.39—carboxylase		2	MSTRG.24551, MSTRG.24552
Biosythesis of antibiotics	ec:4.1.1.39—carboxylase		2	MSTRG.24551, MSTRG.24552
Carbon fixation in photosynthetic organisms	ec:4.1.1.39—carboxylase		2	MSTRG.24551, MSTRG.24552
Thiamine metabolism	ec:3.6.1.15—phosphatase		2	MSTRG.24512, MSTRG.24554
Pyrimidine metabolism	ec:2.7.7.6—RNA polymerase		1	MSTRG.24544
Galactose metabolism	ec:3.2.1.26—invertase		1	MSTRG.24512
Phenylpropanoid biosynthesis	ec:1.11.1.7—lactoperoxidase		1	MSTRG.24516
Starch and sucrose metabolism	ec:3.2.1.26—invertase		1	MSTRG.24512
	ec:3.2.1.48—α-glucosidase		1	MSTRG.24512

NADH: Nicotinamide adenine dinucleotide.

## Supplementary Materials

Supplementary materials can be found at http://www.mdpi.com/1422-0067/19/3/852/s1.
