# Supplementary material for: Transcriptome Analysis Identifies a 140 kb Region of Chromosome 3B Containing Genes Specific to Fusarium Head Blight Resistance in Wheat
Source: Int J Mol Sci. 2018 Mar 14;19(3):852. doi: 10.3390/ijms19030852 (PMC5877713; doi:10.3390/ijms19030852)
Supplement: Supplementary file 1 [file ijms-19-00852-s001.zip › Manuscript to IJMS (2018-01-31).docx]

Type of the Paper (Article)

Transcriptome analysis identifies a 140-kb region of chromosome 3B containing genes specific to Fusarium head blight resistance in wheat

Xin Li ^1,2,^*, Meng Xu ^1,^*, Feiquan Tan 1, Wanquan Chen ^2,^**, Syeda Akash Fatima ^1^, Qianglan Huang ^1^, Qing Li ^1,3^, Taiguo Liu ^2^, Shengfu Zhong ^1^, Min Zhang ^1^, Huaiyu Zhang ^1^, Shengwen Tang ^1^, Li Lei ^4^ and Peigao Luo ^1,^ ^2,^**

^1^ Provincial Key Laboratory of Plant Breeding and Genetics, Sichuan Agricultural University, Chengdu, Sichuan 611130, China.

^2^ State Key Laboratory for Biology of Plant Diseases and Insect Pests, Institute of Plant Protection, Chinese Academy of Agricultural Sciences, Beijing 100193, China.

^3^ Department of Biology and Chemistry, Chongqing Industry and Trade Polytechnic Institute, Fuling District of Chongqing 408000, China.

^4^ Department of Agronomy and Plant Genetics, University of Minnesota, St. Paul, Minnesota 55108, USA

***** These authors contributed equally to this work

****** Corresponding authors: Peigao Luo (e-mail: [lpglab@](mailto:lpglab@)sicau.edu.cn; Tel: 86-28-86290978; Fax: 86-28-86290870) ; Wanquan Chen (e-mail: wqchen112@ippcaas.cn)

**Abstract:** Fusarium head blight (FHB), mainly caused by *Fusarium graminearum*, is one of the most destructive fungal diseases of wheat (*Triticum aestivum* L.). Due to the quantitative nature of FHB resistance, its mechanism is poorly understood. We conducted a comparative transcriptome analysis to identify genes that are differentially expressed between FHB-resistant and FHB-susceptible wheat lines grown under field conditions at various periods after *F. graminearum* infection and determined the chromosomal distribution of the differentially expressed genes (DEGs). For each line, expression in the spike (which exhibits symptoms in infected plants) was compared with that in the flag leaves (which do not exhibit symptoms in infected plants). We identified an island of 53 constitutive DEGs in a 140-kb region with high homology to the *FhbL693b* region on chromosome 3B. Of these genes, 13 were assigned to specific chloroplast-related pathways. Furthermore, one encoded inositol monophosphate (IMPa) and two encoded ribulose-1,5-bisphosphate carboxylase/oxygenase (RuBisCO).

Our findings suggest that the temporary susceptibility in locally infected spikes results from crosstalk between RuBisCO and IMPa, which blocks secondary signaling pathways mediated by salicylic acid and induces systemic acquired resistance in distant leaf tissue.

**Keywords:** Fusarium head blight; Gene island; Photosynthesis; Transcriptome; Wheat.

1. Introduction

Wheat (*Triticum aestivum* L.) is the most widely grown crop worldwide and accounts for approximately 20% of the calories consumed by humankind [1]. Wheat Fusarium head blight (FHB), also called ‘wheat cancer’ or ‘scab’, is an economically important fungal disease that is mainly caused by *Fusarium graminearum.* FHB seriously threatens wheat production around the world [2]. The accumulation of mycotoxins produced by Fusarium, especially deoxynivalenol, in wheat and wheat products, causes acute food poisoning in humans, and harms animals that consume the infected grain [3,4].

While crop management practices and chemical applications may reduce the damage caused by FHB, developing resistant cultivars is critical for combating this disease [5,6]. The level of resistance to FHB in wheat cultivars is low, even among cultivars that are less susceptible to FHB, such as Sumai 3 and Wangshuibai. To date, no genes have been characterized that confer complete resistance to FHB in any cultivar, and this has impeded the breeding of resistant wheat [7]. The molecular mechanism underlying plant defense against *F. graminearum* infection is unknown. From both economic and human health perspectives, enhancing FHB resistance in wheat is critical for reducing yield loss and mycotoxin contamination.

FHB resistance is quantitative in nature, involving the additive effects of several genes [8]. The genetic factors underlying resistance to FHB are also highly complex, and experimental errors may have masked differences in resistance levels among genotypes [9]. The development of resistant cultivars has been impeded because of our poor understanding of the genetic mechanisms of FHB resistance. Gene expression profile changes have provided key insights into the genetic mechanisms involved in pathogen invasion. Recently, six putative FHB resistance genes were localized within the *Fhb2* region of chromosome 6B using transcriptome analysis [10]. Thus, transcriptome analysis is a useful tool for elucidating the genetic basis of this complex trait.

In addition to causing symptoms in the spike, FHB seriously affects developing seeds. Since developing seeds are the most important carbohydrate sinks, and there is a dynamic balance between carbohydrate sources (e.g., the leaves) and sinks [11,12], changes in gene expression in various tissues would be expected to occur upon plant pathogen attack [13]. Therefore, it would be interesting to characterize changes in gene expression in both the leaves and the spikes of wheat plants during FHB infection, and to use the expression levels in the leaves as a control when considering gene expression changes in the spike.

Genotypes with high resistance and simple resistance mechanisms provide a valuable pool of genes to improve crop resistance, and are a prerequisite for fully understanding the resistance mechanism. In the past, studies of FHB resistance have focused on two Chinese wheat landraces, Wangshubai and Sumai 3, and their derived offspring. It is important to identify new sources of resistance against FHB in wheat, both to elucidate the resistance mechanisms and to improve cultivars.

In a previous study, we developed and identified a wheat line L693 from F_6:7_ families of a cross between wheat cultivar MY11 and the line YU25, as the donor of three disease resistance genes [14,15] that exhibit excellent resistance to stripe rust [14,16,17], powdery mildew [17], and FHB [17-19]. The sister line L661, derived from the same F_6:7_ families, was susceptible to stripe rust and FHB [17,18]. Further molecular tests revealed that L693 and L661 had highly similar genetic backgrounds in which 41 (2.4%) polymorphic loci were shared among 1703 loci amplified by 781 simple sequence repeat (SSR) primers [16]. This segregation in resistance showed that resistance in F_2_ and F_2:3_ populations was inherited as two Mendelian factors, *FhbL693a* and *FhbL693b.* We then detected two major quantitative trait loci (QTLs), *Qfhs-2B* and *Qfhs-3B*, associated with FHB resistance [20]. Information regarding pedigree, inheritance, resistance response, chromosomal location, and marker diagnosis indicated that *Qfhs-3B* was different from *Fhb1*; it behaved as a single Mendelian factor and was given the gene symbol *FhbL693b* [20].

L693 is clearly an important source for disease resistance in wheat breeding programs. However, despite the successful detection and mapping of QTLs for FHB resistance in L693, the underlying molecular basis of FHB resistance remains to be elucidated.

The objective of the present study was to explore the molecular basis of FHB resistance in wheat. Two wheat genotypes, consisting of sister lines L693 (resistant) and L661 (susceptible) were inoculated with *F.* *graminearum*. As differences caused by genetic background were minimized in these lines, we could easily identify key resistance (R) genes and the chromosomal regions responsible for FHB resistance. The expression of several randomly chosen genes in the spike (inoculated tissue; local tissue) and leaf (non-inoculated; control, distant tissue) was profiled using RNA-sequencing (RNA-Seq) and confirmed using quantitative real-time PCR (qPCR). Differentially expressed genes (DEGs), and their chromosomal distributions were compared between the two genotypes. Furthermore, we compared gene expression dynamics between the inoculated spike and non-inoculated leaf between the two genotypes, with the aim of identifying the functions and metabolic pathways of genes in the *FhbL693b* chromosomal region that are differentially expressed in the spikes and leaves of the two lines following fungal infection.

Our results provide insight into the genetic basis of FHB resistance in L693, and suggest a molecular strategy for FHB resistance based on global expression profiling. Furthermore, this work identifies potential genes for breeding applications.

2. Results

2.1. L693 and L661 exhibit different FHB resistance levels, but similar transcriptome sizes

We found that L693 exhibited a high level of FHB resistance [20]; only the inoculated spikelet had dried out and died at 21 days after inoculation (DAI) with *F. graminearum*. By contrast, in L661, after the same period of time, almost the entire spike containing the inoculated spikelet had dried out and died (Fig. 1a). Furthermore, grains of L693 appeared fully filled at 21 DAI, as normal, while the grains of L661 were shriveled (Fig. 1b). The percentage of diseased spikes (PDS) was significantly higher (p<0.01), based on multiple comparisons, in L661 than in L693 (Fig. 1c).

RNA samples were taken from spike and leaf tissues of the resistant line L693 and the susceptible line L661 at three infection stages: 0, 24, and 72 hours after inoculation (HAI) with *F. graminearum*, and analyzed (Table 1). Genes expressed in at least 3 out of 12 samples were considered expressed genes. Of the 47 443 expressed genes identified, 2453, 2103, and 399 genes in the L693 spike were upregulated compared with the L661 spike, whereas 644, 754, and 374 genes were downregulated at 0, 24, and 72 HAI, respectively (Fig. 2a). However, in leaf tissue, 914 genes were downregulated and 1192 and 375 genes were upregulated in L693, compared to 966 genes that were up-regulated, and 842 and 311 that were downregulated in L661 at 0, 24, and 72 HAI (Fig. 2b), respectively.

2.2. Validation of differences in gene expression by RT-qPCR and clustering analysis

To validate the differential gene expression of the 12 samples analyzed (i.e., two genotypes; two tissues; three time points), five genes were chosen randomly for RT-qPCR analysis (Table S1). The correlation between normalized mRNA-seq RPKM results and qRT-PCR expression values was high (R^2^ = 0.73) (Fig. 3a, Supplementary Material 2: Table S1). In addition, 12 clustering samples based on Illumina read counts mapped against the Ensembl wheat genome sequence showed that the tissue type had the biggest effect on changes in gene expression, followed by the time after inoculation, followed by the genotype of wheat used (Fig. 3b). Clustering results showed that the difference in gene expression between the spike and leaf was greater than that between genotypes.

2.3. Differential expression analysis revealed tissue-specific expression tendencies

Using edgeR software [21], mor**e** DEGs were identified in the spike than in the leaf of both L693 and L661 plants. Among the 3097 DEGs identified in the spike at 0 HAI, 14 were strongly upregulated (i.e., had logFC values of greater than +12), but only two were strongly downregulated (i.e., with logFC values of less than –12). High expression abundance (i.e., with log-counts per million logCPM values of greater than 10) was found in 126 of 2453 upregulated genes (shown in blue in Fig. 4a) compared to 644 downregulated genes. At 24 HAI, there were 2857 DEGs in the spike, including 13 that were strongly upregulated and only one that was strongly downregulated in L693 compared to L661 (Fig. 2a, Supplementary Material 1: Fig. 4b, Supplementary Material 2: Table S2). At 72 HAI, 773 DEGs were observed in the spikes of the two genotypes, including seven that were strongly upregulated and three that were strongly downregulated in L693 compared to L661 (Fig. 2a, Supplementary Material 1: Fig. 4c, Supplementary Material 2: Table S2). Seven of the 374 downregulated genes had higher logCPM values than those of 399 upregulated genes.

In leaf tissues, there were 1880 DEGs at 0 HAI between the two genotypes; these included 11 strongly up-regulated genes and six strongly downregulated genes in L693 compared to L661. Forty-eight of 914 upregulated genes had high expression abundance compared to 966 downregulated genes. At 24 HAI, there were 2034 DEGs between the two genotypes, including 10 that were strongly upregulated and three that were strongly downregulated in L693 compared to L661. At 72 HAI, 686 DEGs were detected between the two genotypes, only two of which were strongly upregulated and one of which was strongly downregulated (Fig. 2b, Supplementary Material 1: Fig. 4f, Supplementary Material 2: Table S2).

We identified some similar changes in gene expression between the spike and leaf tissues (Fig. S1). The number of upregulated genes in the spike compared to the corresponding leaf was greater than of downregulated genes at each time point in both L693 and L661 plants. The change in gene expression in the L661 spike from 0 to 24 HAI was greater than that of L693 (Supplementary Material 1: Fig. S2d, j). By contrast, there was a greater change in gene expression in the L693 spike compared to L661, both from 0 to 72 HAI and from 24 to 72 HAI (Supplementary Material 1: Fig. S2e, f, k, l). Many of the upregulated genes in the L661 spike from 0–24 HAI and 0–72 HAI had larger logCPM values than the corresponding downregulated genes (Supplementary Material 1: Fig. S2 d, e). A greater number of DEGs were seen in the L693 spike from 24 to 72 HAI than in the L661 spike (Supplementary Material 1: Fig. S2 f, l). Moreover, in L693, there were more down-regulated genes than upregulated genes, while in L661 there were fewer downregulated genes than upregulated genes (Supplementary Material 2: Table S3). Expression of genes in the leaves of both genotypes were similar; the largest difference between L693 and L661 spikes occurred from 0 to 24 HAI, while that in leaves was from 24 to 72 HAI (Supplementary Material 1: Fig. S2).

2.4. DEGs have a biased chromosomal distribution

The chromosomal distribution of DEGs was biased (Fig. 5). In the spike, 36 (10.8%) of 333 genes differentially co-expressed between L693 and L661 at the three time points examined were mapped to chromosome 3B. In the leaf, 33 (11.1%) of 298 genes differentially co-expressed between L693 and L661 at the three time points were mapped to chromosome 3B. In addition, there was a similar number of total DEGs and DEGs on chromosome 3B between L693 and L661 at the three time points (Fig. 5, Supplementary Material 2: Table S4). Genes that were differentially co-expressed in the spike and leaf at three points in both L693 and L661 exhibited no biased distribution on chromosome 3B (Supplementary Material 1: Fig. S3). In both L693 and L661 spikes, no biased distribution of co-expressed DEGs (24 vs 0, 72 vs 0 and 72 vs 24) on chromosome 3B was found, but the number of co-expressed DEGs (24 vs 0, 72 vs 0 and 72 vs 24) mapping to chromosome 3B in the leaves of L693 and L661 plants at the three time points was only 9 (2.8%) and 15 (3%) out of 317 and 496, respectively, which is fewer than the chromosomal average value of about 5.2% (Supplementary Material 1: Fig.S4, Supplementary Material 2: Table S4).

2.5. A 140-kb differential expression island exists on chromosome 3B

We then searched sequence data (http://plants.ensembl.org/Triticum_aestivum) for genes on wheat chromosome 3B. We identified 8571 annotated genes with a total length of 774 434 471 bp, excluding scaffolds, and an average length of 90.4 kb. Further analysis of the reference sequence revealed a gene island in the region between 181.40 and 181.54 Mb; this 140-kb sequence contained 73 annotated genes with an average density of 1.9 kb. Reliable transcription products of 1350 (16.8%) out of 8571 annotated genes were detected with an average density of 573.7 kb, while 42 (57.5%) out of 73 annotated genes in the 3B chromosomal region from 181.40 to 181.54 Mb were transcribed. RNA-Seq data led to the identification of 4136 unannotated transcribed genes that may encode unknown RNAs or proteins; these genes are hereafter referred to as novel transcribed genes (NTGs). A total of 12 707 annotated genes (including previously annotated genes and NTGs) were identified on chromosome 3B with an average gene density of 60.9 kb, while 86 genes were found on chromosome 3B from 181.40 to 181.54 Mb, with an average gene density of 1.6 kb (Fig. 6). Furthermore, 2456 (19.3%) out of 12 707 genes on chromosome 3B exhibited reliable expression with an average gene density of 315.3 kb, while 53 (61.6%) out of 86 genes were reliably expressed on chromosome 3B from 181.40 to 181.54 Mb, with an average gene density of 2.6 kb.

2.6. Genes in the 140-kb expression island exhibit higher constitutive expression in the FHB resistance genotype

Twenty-seven out of 53 genes had a higher expression level (i.e. –2 < logFC < 2) in the L693 spike than in the L661 spike at 0 HAI, while no difference in gene expression was observed (i.e. –2 < logFC < 2) between the spikes of L693 and L661 at 24 or 72 HAI. In the leaf, 9, 2, and 1 out of the 53 genes were differentially expressed between L693 and L661 at 0, 24, and 72 hours, respectively. In all differentially expressed genes, the expression level in L693 was visibly higher than that in L661. Furthermore, in both the spike and leaf, all 36 genes expressed in L693 and L661 at 0 HAI were upregulated in L661 from 0 to 24 HAI, while only 24 genes were upregulated in L693 during that period (Fig. 7, Supplementary Material 2: Table S5).

2.7. DEGs in the 140-kb expression island are mainly involved in chloroplast function

Of the 53 DEGs identified, 42 were previously annotated and 11 (with transcript ID name ‘MSTRG’ in Table S5) were newly annotated in this study. Using the BLAST program, DEGs were identified that had high levels of nucleotide sequence similarity with annotated genes for chloroplast-related pathways (39; 73.6%), mitochondrion-related pathways (3; 5.7%), and genes encoding putative proteins without known functions (7; 13.2%). Four (7.5%) DEGs showed no similarity to any previously identified genes (Table S5). Further Kyoto Encyclopedia of Genes and Genomes (KEGG) pathway enrichment analysis revealed that 13 (24.5%) of the 53 DEGs were assigned to a specific pathway (Supplementary Material 2: Table S5); of these, 7 (53.8%) were involved in oxidative phosphorylation pathways (Supplementary Material 2: Table 2). Some DEGs were involved in other known pathways, including purine metabolism, glyoxylate and dicarboxylate metabolism, biosynthesis of antibiotics, carbon fixation in photosynthetic organisms, thiamine metabolism, pyrimidine metabolism, galactose metabolism, phenylpropanoid biosynthesis, and starch and sucrose metabolism (Supplementary Material 2: Table S5). Thirteen DEGs showed wide pleiotropism; for example, *MSTRG.24512,* which encodes ec:3.6.1.3-adenylpyrophosphatase and ec:3.6.1.15-phosphatase, participates in four pathways including purine metabolism, thiamine metabolism, galactose metabolism, and starch and sucrose metabolism (Supplementary Material 2: Table S5). Eleven (84.6%) of these 13 DEGs are involved in chloroplast-related metabolism, and 2 of these DEGs are involved in mitochondrion-related metabolism. Further analysis found that 9 and 4 of the 13 DEGs were differentially expressed between the two genotypes at 0 DAI in the spike and leaf, respectively; with line L693 exhibiting higher constitutive expression than line L661. We paid specific attention to three interesting constitutively expressed genes that were induced upon inoculation with *F. graminearum*: one encoding inositol monophosphate (IMPa) and two encoding ribulose-1,5-bisphosphate carboxylase/oxygenase (RuBisCO).

3. Discussion

In this study, we used RNA-Seq to characterize genes that were differentially expressed in the resistant the L693 genotype, which carries *FhbL693b,* and the susceptible L661 genotype, which lacks *FhbL693b*, following *F. graminearum* infection and compared the expression of genes in the spikes and leaves of both lines at various time points. Our findings pave the way for developing an integrated model for explaining the response to *F. graminearum* infection in wheat.

3.1. The reliability of original data was important for comparing transcriptome analyses

We collected RNA-Seq samples at three time points (0, 24, and 72 hai) from two genotypes (L693 and L661) and two tissues (spike and leaf), with the aim of analyzing the effect of genotype, tissue type, and time on gene expression changes. No mock inoculation was performed, as this would not have provided any additional information in the complex environment of the field, but would have increased the cost of the sequencing. As gene expression analysis is considered to be equally accurate in the field and the greenhouse [22], we performed all of the inoculations and sample harvests under field conditions.

The difference in the PDS was significant between the two genotypes, but was not affected by the experimental conditions used (i.e., 2013 vs. 2014; field vs. greenhouse) (Fig. 1c), which indicated that infection in the field was as effective as in the greenhouse. After infection, the change in gene expression exhibited a similar tendency and degree between the L693 and L661 lines (Fig. S1, Supplementary Material 1: Fig. S2). Further analysis of 12 samples showed that the greatest difference in gene expression following *F. graminearum* infection occurred between different tissues (spike vs. leaf), while the smallest difference was observed between the resistant and susceptible genotypes (Fig. 3b). This could be due to the fact that L693 and L661 have similar genetic backgrounds [16,20,23]. The fact that the greatest difference in gene expression occurred between the spike and leaf indicated that the transcriptome data were reliable to some degree, which could be a real response after *F. graminearum* infection. To further validate the data, we randomly selected 5 genes for RT-qPCR analysis (Supplementary Material 2: Table S1). A high correlation (R^2^ = 0.73) between RPKM and RT-qPCR expression values (Fig. 3 Supplementary Material 2: Table S1) indicated that the RNA-seq data were indeed reliable.

3.2. DEGs in both the leaf and spike play key roles in FHB resistance formation

Following pathogen attack, plants must redistribute energy and resources from primary metabolism to the induced reaction [24]. Previous studies of wheat spike diseases such as FHB have usually focused on gene expression changes only in local spike tissue, whereas studies of wheat leaf diseases, such as stripe rust and powdery mildew, have focused on gene expression changes only in the local leaf tissue [13,23]. Gene expression changes in distant tissues of plants infected by pathogens, such as the leaves of wheat plants infected by *F. graminearum,* have not been documented.

In the present study, a comparison of the L693 and L661 lines showed that more genes were upregulated than downregulated in the spike after *F. graminearum* infection, while a similar number of genes were expressed in the leaves of these lines at 0 h (Fig. 2a). Interestingly, the spike exhibited fewer upregulated genes than downregulated genes, while the number of genes expressed in the leaf increased at 24 h. At 72 h, the number of upregulated genes was almost equal to that of downregulated genes in both the spike and leaf (Fig. 2a, Fig. 4). Many genes that were constitutively expressed at higher levels in the spike were induced in the leaves of the FHB-resistant genotype, but not in those of the FHB-susceptible genotype. Though the pore-forming toxin-like (PFT) gene at *Fhb1* was also constitutively expressed in the spike of the FHB-resistant genotype, the expression in uninoculated leaves was not determined [25]. As FHB resistance mainly results from the systemic acquired resistance induced by infection of the pathogen, we propose that the strongly induced gene expression observed in the leaf may play an important role in FHB resistance.

3.3. The gene island and 140-kb differential gene expression island on chromosome 3B were highly associated with FHB resistance

A gene island is a gene-rich genomic region [26]. Previous studies demonstrated that gene islands are abundant in the wheat genome [27-29]. In this study, we firstly found that genes that were differentially expressed following *F. graminearum* infection exhibited an obvious bias in chromosomal location to chromosome 3B (Fig. 5, 6, Table 2). In both the spike and leaf, differential co-expressed genes on chromosome 3B between the L693 and L661 lines represented more than 10% of the total number of differential co-expressed genes at the three time points (Table 2). Furthermore, in both L693 and L661 leaves, the differential co-expressed genes on chromosome 3B represented 3% or less than the total number of differential co-expressed genes at each time point (Supplementary Material 1: Fig. S3, Table 2), indicating that the differential gene expression on chromosome 3B was highly related to FHB resistance. Secondly, a gene island from 181.40 to 181.54 Mb was identified. The average gene length within the gene island was 1.6 kb, whereas it was 60.9 kb on the whole of chromosome 3B. Thirdly, the region from 181.40 to 181.54 Mb of chromosome 3B was also a gene expression island, in which a proportion (61.6%) of the expressed genes was markedly higher than that (19.3%) on the whole of chromosome 3B. Thus, a gene island and a gene expression island exist in region 181.40 to 181.54 Mb of chromosome 3B.

*FhbL693b* was different from the well-known FHB resistance QTL *Fhb1* on 3BS, based on genetic mapping results [20]. The chromosomal region from 181.40 to 181.54 Mb was also different from the *Fhb1* region in view of physical mapping [25]. In addition, the gene island from the wheat reference sequence and gene expression island from the transcriptome were located within the FHB resistance QTL *FhbL693b*. Together, these findings indicate that the gene island and gene expression island in the chromosomal region from 181.40 to 181.54 Mb were functional FHB resistance gene islands, which could mediate FHB resistance both via genomic constitutive expression and induced expression after infection by *F. graminearum*.

3.4. Constitutive differential expression of genes in the 181.40 to 181.54 Mb region of chromosome 3B plays a key role in FHB resistance

Many genes in the chromosomal region from 181.40 to 181.54 Mb exhibited markedly higher constitutive expression in both the spike and flag leaf of L693 as compared to L661 (Supplementary Material 2: Table S5). We found that 27 and 9 DEGs exhibited markedly higher constitutive expression in the spike and flag leaf, respectively, of L693 plants than of L661 plants (Supplementary Material 2: Table S5). Interestingly, all 53 DEGs were upregulated in the spike of L661 at 24 HAI as compared to 0 HAI, and 41 of these were markedly upregulated. However, in the spike of L693 plants, only 12 of the 39 upregulated genes were markedly upregulated, and 1 gene exhibited downregulated expression (Supplementary Material 2: Table S5). These observations indicated that the higher constitutive expression of these genes in region 181.40 to 181.54 Mb of chromosome 3B, especially in the spike, might play a key role in FHB resistance. A recent publication reported the pore-forming toxin-like (PFT) gene as a candidate gene of *Fhb1* exhibiting a higher constitutive expression in R-NIL, resulting in a sharp decline in transcript levels after emergence of spikes [25], which further supports our proposal that increased constitutive expression plays a vital role in FHB resistance.

3.5. Photosynthesis is possibly involved in FHB resistance

The chloroplast is involved in energy production, redox homeostasis, and retrograde signaling, and these processes collectively participate in the plant immune response [30]. The plant’s response to pathogen attack is closely linked to a change in energy metabolic pathways, such as photosynthesis [31]; therefore, the process could be tracked and quantified using photosynthesis-related parameters, including net photosynthetic rate and chlorophyll fluorescence [19,32,33]. Historically, immunity and photosynthesis were studied separately. Therefore, to discuss the crosstalk between photosynthesis and immunity would be useful for plant protection. In the present study, we found that 13 (24.5%) out of 53 DEGs were assigned to the specific pathway and 7 (53.8%) out of 13 genes in region 181.40 to 181.54 Mb of chromosome 3B were involved in oxidative phosphorylation pathways (Supplementary Material 2: Table S5). We also found that specific metabolic pathways of 11 genes occur in the chloroplast and only two, encoding the apocytochrome b genes MSTRG.24508 and MSTRG.24508, occur in the mitochondrion (Supplementary Material 2: Table S5), indicating that photosynthesis in both the spike and leaf could play a vital role in regulating wheat resistance to FHB.

By conducting a comparison of differential constitutive gene expression and induced changes in expression following *F. graminearum* infection between two genotypes, we further identified three intriguing genes: MSTRG24516 (encoding inositol monophosphatase (IMPa)) and MSTRG24551 and MSTRG24552 (encoding ribulose-1,5-bisposphate carboxylase/oxygenase, RuBisCO). IMP biosynthesis catalyzed by IMPa is crucial in multicellular eukaryotes, while IMPa is required for the breakdown of inositol (1,4,5)-trisphosphate, an important secondary messenger involved in Ca^2+^ signaling [34,35]. A recent study demonstrated that IMP inhibited the salicylic acid-dependent pathogen defense responses triggered by reactive oxygen species (ROS) [36]. This information supports our hypothesis that the differential expression of MSTRAG24516 is involved in wheat FHB resistance.

In addition, RuBisCO acts as a key regulator that controls the balance between photosynthesis and photorespiration. An oxidative burst mainly resulting from an increase in photorespiration during primary fungal pathogen infection produces various ROS [37], which negatively influences resistance or promotes disease development [38]. *F. graminearum* infection can be divided into biotrophic and necrotrophic stages, and therefore, it uses both living and dead tissue for nutritional purposes, which causes the host to be less resistant to FHB than to other wheat diseases. Tissue necrosis caused by the primary ROS during pathogen infection increases wheat host susceptibility to the necrotrophic form of *F. graminearum*, but resistance to its biotrophic form. On the one hand, pathogen-induced ROS are signaling molecules that trigger systemic resistance. This type of resistance is characterized by the rapid generation of hydrogen peroxide (H_2_O_2_), which acts as a secondary messenger that induces the expression of defense genes [39]. On the other hand, ROS could directly act as a weapon to resist pathogens, because these molecules are also harmful to pathogens [40]. In this study, there was markedly higher constitutive expression of MSTRG24551 and MSTRG24552 in both the spike and leaf of L693 as compared to L661, indicating that MSTRG24551 and MSTRG24552, which encode RuBisCO, are also involved in wheat FHB resistance

To further elucidate the crosstalk between IMP and RuBisCO, it is useful to understand their mechanisms of action in the secondary signaling pathway. In the spikes of L693, but not L661, plants, we observed a sharp decrease in RuBisCO expression following local infection (Supplementary Material 2: Table S5), and this decrease was accompanied by an increase in net photosynthetic rate (Pn) (Supplementary Material 2: Fig. S5), indicating that photorespiration could be blocked to some degree, resulting in less oxidative stress. Less oxidative stress not only has a positive effect on IMP biosynthesis, but also possibly blocks the plant resistance signaling pathway mediated by SA. Interaction between IMP and RuBisCO may lead to temporary susceptibility and short-lived resistance in the locally infected spikes of L693 and L661 plants, respectively, following primary physiological signaling that triggers the secondary signaling pathway in distant tissue such as the leaf. In the distant uninfected leaves of L693 plants, the increased RuBisCO (Supplementary Material 2: Table S5), as compared to L661, was used for photorespiration, as there was a decrease in *P_n_* (Supplementary Material 1: Fig. S5a), causing high oxidative stress, which contributed to the secondary signaling pathway mediated by SA, and this produced systemic acquired resistance against wheat FHB. Comparisons of SOD and CAT contents in the two lines over time (Supplementary Material 1: Fig. S 5b and 5c) would support our assertion. Additionally, the chloroplast protein NRIP1, which is responsible for Tobacco mosaic virus recognition [41]; the Arabidopsis protein PHYTOPHTHORA1, which imparts resistance to *Phytophthora brassicae* [42]; and the wheat kinase START1 resistance protein (WKS1) [43] are localized to the chloroplast, supporting the view that RuBisCO is also involved in FHB resistance.

In conclusion, changes in the balance between photosynthesis and photorespiration regulated by RuBisCO could lead to low ROS and/or high IMP levels (Fig. 8), both of which would block secondary signaling pathway mediated by SA, causing brief susceptibility in the local spike of FHB-resistant L693. Subsequently, the short susceptible response would induce distant leaf tissue to produce systemic acquired resistance by opposite regulation of the above pathways mediated by SA .

4. Materials and methods

4.1. Plant and pathogen materials

FHB-resistant wheat line L693 and FHB-susceptible line L661 were selected and developed from F_6:7_ families derived from a cross between the FHB-susceptible wheat cultivar MY11 and FHB-resistant line YU25 [17,18,20]. Several years of continuous resistance testing confirmed that L693 was strongly, stably, and consistently resistant to FHB [17,18]. Further studies showed that FHB resistance in all tested populations was inherited as two Mendelian factors, *FhblL693a* and *FhbL693b* [20]; therefore, this line was selected for further study of the FHB resistance mechanism. The monosporic isolate of *F. graminearum* Fg 4 was used for fungal infection; this was kindly provided by Professor Ma Zhengqiang (Nanjing Agricultural University, Nanjing, Jiangsu Province, China).

4.2. F. graminearum spore production and inoculation

A macroconidial suspension was produced and harvested as previously described [19]. Wheat spikes from L693 and L661 were point-inoculated by injection with freshly prepared spore suspension at the anthesis stage, and four florets of two central spikelets per spike were used. Each floret was injected with 5 µl of macroconidial suspension (200 macroconidia per µl), and was covered by a plastic bag to maintain humidity; bags were removed 72 h after inoculation. Whole spikes and flag leaves of two genotypes were harvested at 0 h (non-inoculated), 24 h, and 72 h, respectively.

4.3. Experimental design and sample harvesting

To understand the mechanism of FHB resistance under natural field conditions, seeds of L693 and L661 were grown in fields at the Wenjiang Experimental Station of Sichuan Agricultural University (latitude 30° 43ʹN, longitude 103° 52ʹN), in southwest China during the 2012–2013 wheat growing season, which was temperate and rainy (annual average temperature of 17°C and rainfall of 1350–1580 mm). Field experiments used a randomized complete block design with three replications. Each block consisted of twelve 3-m-long rows (with a row spacing of 33 cm), with an interplant width of 15 cm. Plants randomly chosen from each population, which were distributed across three blocks, were marked at spike emergence (about March 21, 2013) for future inoculation. Previously described methods were used to control leaf diseases such as stripe rust and powdery mildew, and for fertilizer and pest management [12]. Inoculation of samples was started at 9 am. To detect gene expression change in the inoculated local spike, expression levels were compared with those in a distal non-inoculated leaf. The 0 h samples (non-inoculated) were used as a control. Therefore, 12 samples (two genotypes, two tissues, and three time points) were prepared for RNA extraction per block. FHB resistance was also evaluated in plants grown in the greenhouse at Kansas State University (Manhattan, Kansas 66506, USA) according to a previously described program [17].

4.4. RNA extraction and RNA-sequencing

Total RNA was isolated using TRIzol reagent (Invitrogen) according to the manufacturer’s protocol. The total RNA of three leaves and three spikes of the same wheat line from each block at the same time point were mixed in equal amounts for transcriptome sequencing. A transcriptome library with fragments ranging in size from 200–700 bp was prepared using an Illumina Kit, and sequenced on an Illumina HiSeq™ 2000 using paired-end technology in a single run. Single-end sequencing ensured that the sequencing depth of each sample exceeded 5 Gb. RNA-Seq was executed by ABlife Inc. (Wuhan, China).

4.5. Reliable analysis of sequencing data and quantitative real-time PCR

Raw reads were trimmed if they contained more than 2-N bases, and then processed by adaptor clipping and removal of low quality bases. Short reads of less than 16 nt in length were also removed. FASTX-Toolkit (v 0.0.13) was used to obtain clean reads, and Fastqc (http://www.bioinformatics.babraham.ac.uk/projects/fastqc/) was used to check the quality of clean reads. To further demonstrate the reliability of the RNA-Seq data, 5 genes were chosen randomly from the expressed genes for quantitative PCR (qPCR) experiments. Details of the primer sequences and descriptions of the genes are shown in (Supplementary Material 2: Table S1). Complementary DNA (cDNA) for real-time quantitative reverse transcription PCR (qRT-PCR) was synthesized by M-MLV (Invitrogen). Each 20 µl reaction contained 4 µg RNA, 2 µl cDNA diluted with pure water (1:20), and 9 µl RealMasterMix (SYBR solution; TIGEN). The MiniOpticon Time PCR Detection System was used for qPCR as previously described [23].

4.6. Differential gene expression analysis and identification of hotspot expression polymorphism

To determine expression polymorphism, uniquely mapped reads were counted using Bowtie and RSEM software [44] and the values of reads per kb per million mapped sequence reads (RPKM) were computed as previously described [45]. Differential gene expression between the spike and leaf of L693 or L661 at various time points was measured using TMM and DEGseq software [46]. The difference in fold-change for unigenes was computed as described [47]. DEGs were mapped to the reference genome using BOWTIE2[48]. Based on the peaks of DEGs on each chromosome, transcript hotspots of expression polymorphism were identified, where a polymorphism caused differential accumulation of a specific transcript in the genome [49].

4.7. Bioinformatic analysis of pathway-specific gene function in the FhbL693b region

Identified unigenes were analyzed using BLASTX and BLASTN. Putative physiological and functional categories were assigned as per the GoFigure Gene Ontology (GO) annotation [12].

**Supplementary Materials:** Supplementary materials can be found at www.mdpi.com/link.

**Supplementary Material 1: Figure S1.** Log-fold change (logFC) against log-counts per million in different tissues. Differentially expressed genes with an FDR of <5% and a log-fold change of >2 are highlighted in red. In each comparison, red dots below the blue line (logFC < 2) representing up regulated genes in leaf or down regulated genes in spike, in opposite, red dots above the blue line (logFC > 2) representing up regulated genes in spike or down regulated genes in leaf. The blue dots representing gene(s) with extreme value with -12 > logFC > 12 or have Average logCPM more than 10. The green dots represent the interested gene in this study, rectangle for MSTRG.24516, circle for MSTRG.24551, triangle for MSTRG.24552. **Figure S2.** Log-fold change (logFC) against log-counts per million at different time points. Differentially expressed genes with an FDR of <5% and a log-fold change of >2 are highlighted in red. The blue dots representing gene(s) with extreme value with -12 > logFC > 12 or have Average logCPM more than 10. The green dots represent the interested gene in this study, rectangle for MSTRG.24516, circle for MSTRG.24551, triangle for MSTRG.24552. **Figure S3.** Venn diagram displaying the number of differentially expressed genes between the leaves and spikes of L693 (**A**) and L661 (**B**) plants at 0 h (red), 24h (green), and 72 h (blue). Numbers without parenthesis stand for different expressed genes over the whole genome, numbers with parenthesis stand for different expressed genes on wheat chromosome 3B. **Figure S4.** Venn diagram displaying the number of differentially expressed genes in the L693 spike (**A**), L693 leaf (**B**), L661 spike (**C**), and L661 leaf (**D**) at 0 h and 24 h (red), 0 h and 72 h (green), and 24 h and 7 2h (blue). Numbers without parenthesis stand for different expressed genes over the whole genome, numbers with parenthesis stand for different expressed genes on wheat chromosome 3B. **Figure S5.** Changes in net photosynthetic rate (*P_n_*) in the leaf (**A**) and spike (**B**), antioxidant enzyme activity (SOD) in the leaf (**C**) and spike (**D**), and catalase activity (CAT) in the leaf (**E**) and spike (**F**) following *Fusarium graminearum* inoculation.

**Supplementary Material 2: Table S1.** mRNA-seq RPKM and qRT-PCR expression values of five randomly choosen genes. **Table S2.** List of differentially expressed genes coresponding to Fig. 4. **Table S3.** List of differentially expressed genes coresponding to Fig. S2. **Table S4.** Statistically significant expression biases in chromosome 3B. **Table S5**. Details of 53 genes on regions 181.40–181.54 Mb of wheat chromosome 3B. Genes with colored backgrounds have matches in the Kyoto Encyclopedia of Genes and Genomes (KEGG) pathway database.

**Acknowledgments:** This work was supported by National Natural Science Foundation of China (grant numbers 31271721 and 31571661), the Key Projects of the Education Ministry of China (2012146), the Specific Foundation of Agronomy (nyhyzx3-15, 201303016), the Bureau of Science and Technology of Fuling of Chongqing (FLKJ-2015ABB1048), and the Ministry of Science and Technology, China (2013CB127701). We are grateful to professor Ma Zhengqiang of Nanjing Agricultural University, Nanjing, Jiangsu Province, China for providing us with the monosporic isolate of *F. graminearum* Fg 4.

**Author contributions:** W.Q. Chen, T.G. Liu, Xin Li, and P.G. Luo designed the research; X. Li, M. Xu, Q.L. Huang, Q. Li, S.F. Zhong, and S.W. Tang performed the research; M. Zhang and H.Y. Zhang conducted the field tests; X. Li, M. Xu, S.F Zhong, L. Lei, and P.G. Luo analyzed the data; and X. Li, M. Xu, S.A. Fatima, and P.G. Luo wrote the paper.

**Conflicts of Interest**: The authors declare no conflict of interest.

References

1. Nigro, D.; Giove, S.; Fortunato, S.; Incerti, O.; Zacheo, S.; Blanco, A.; Gadaleta, A. Allelic variation of wheat flour allergens in a collection of wheat genotypes. *J. Chem.* **2014**, *2014*.
2. McMullen, M.; Jones, R.; Gallenberg, D. Scab of wheat and barley: A re-emerging disease of devastating impact. *Plant Dis.* **1997**, *81*, 1340-1348.
3. Zwart, R.S.; Muylle, H.; Van Bockstaele, E.; Roldán-Ruiz, I. Evaluation of genetic diversity of fusarium head blight resistance in european winter wheat. *Theor. Appl. Genet.* **2008**, *117*, 813-828.
4. Gilbert, J.; Haber, S. Overview of some recent research developments in fusarium head blight of wheat. *Can. J. Plant Pathol.* **2013**, *35*, 149-174.
5. Bai, G.; Shaner, G. Management and resistance in wheat and barley to fusarium head blight 1. *Annu. Rev. Phytopathol.* **2004**, *42*, 135-161.
6. Walter, S.; Nicholson, P.; Doohan, F.M. Action and reaction of host and pathogen during fusarium head blight disease. *New Phytol.* **2010**, *185*, 54-66.
7. Oliver, R.; Cai, X.; Xu, S.; Chen, X.; Stack, R. Wheat-alien species derivatives: A novel source of resistance to fusarium head blight in wheat. *Crop Sci.* **2005**, *45*, 1353-1360.
8. Zhang, M.; Zhang, R.; Yang, J.; Luo, P. Identification of a new qtl for fusarium head blight resistance in the wheat genotype “wang shui-bai”. *Mol. Biol. Rep.* **2010**, *37*, 1031-1035.
9. Kumar, A.; Karre, S.; Dhokane, D.; Kage, U.; Hukkeri, S.; Kushalappa, A.C. Real-time quantitative pcr based method for the quantification of fungal biomass to discriminate quantitative resistance in barley and wheat genotypes to fusarium head blight. *J. Cereal Sci.* **2015**, *64*, 16-22.
10. Dhokane, D.; Karre, S.; Kushalappa, A.C.; McCartney, C. Integrated metabolo-transcriptomics reveals fusarium head blight candidate resistance genes in wheat qtl-fhb2. *PLoS One* **2016**, *11*, e0155851.
11. Luo, P.; Zhang, H.; Shu, K.; Wu, X.; Zhang, H.; Ren, Z. The physiological genetic effects of 1bl/1rs translocated chromosome in" stay green" wheat cultivar cn17. *Can. J. Plant Sci.* **2009**, *89*, 1-10.
12. Luo, P.; Deng, K.; Hu, X.; Li, L.; Li, X.; Chen, J.; Zhang, H.; Tang, Z.; Zhang, Y.; Sun, Q. Chloroplast ultrastructure regeneration with protection of photosystem ii is responsible for the functional ‘stay‐green’trait in wheat. *Plant, Cell Environ.* **2013**, *36*, 683-696.
13. Ma, L.; Zhong, S.; Liu, N.; Chen, W.; Liu, T.; Li, X.; Zhang, M.; Ren, Z.; Yang, J.; Luo, P. Gene expression profile and physiological and biochemical characterization of hexaploid wheat inoculated with blumeria graminis f. Sp. Tritici. *Physiol. Mol. Plant Pathol.* **2015**, *90*, 39-48.
14. Luo, P.-G.; Hu, X.-Y.; Chang, Z.-J.; Zhang, M.; Zhang, H.-Q.; Ren, Z.-L. A new stripe rust resistance gene transferred from *thinopyrum intermedium* to hexaploid wheat (*triticum aestivum*). *Phytoprotection* **2009**, *90*, 57-63.
15. Luo, P.; Luo, H.; Chang, Z.; Zhang, H.; Zhang, M.; Ren, Z. Characterization and chromosomal location of pm40 in common wheat: A new gene for resistance to powdery mildew derived from elytrigia intermedium. *Theor. Appl. Genet.* **2009**, *118*, 1059-1064.
16. Huang, Q.; Li, X.; Chen, W.; Xiang, Z.; Zhong, S.; Chang, Z.; Zhang, M.; Zhang, H.; Tan, F.; Ren, Z. Genetic mapping of a putative thinopyrum intermedium-derived stripe rust resistance gene on wheat chromosome 1b. *Theor. Appl. Genet.* **2014**, *127*, 843-853.
17. Liu, Z.; Xu, M.; Xiang, Z.; Li, X.; Chen, W.; Luo, P. Registration of the novel wheat lines l658, l693, l696, and l699, with resistance to fusarium head blight, stripe rust, and powdery mildew. *J. Plant Reg.* **2015**, *9*, 121-124.
18. Zhang, L.; Chang, Z.; Li, X.; Zhang, H.; Ren, Z.; Luo, P. Screen and identification of wheat new resistant germplasms to fusarium head blight. *Zhi Wu Bao Hu XueBao* **2011**, *38*, 569-570.
19. Yang, S.; Li, X.; Chen, W.; Liu, T.; Zhong, S.; Ma, L.; Zhang, M.; Zhang, H.; Yu, D.; Luo, P. Wheat resistance to fusarium head blight is associated with changes in photosynthetic parameters. *Plant Dis.* **2016**, *100*, 847-852.
20. Li, X.; Xiang, Z.; Chen, W.; Huang, Q.; Liu, T.; Li, Q.; Zhong, S.; Zhang, M.; Guo, J.; Lei, L. Reevaluation of two quantitative trait loci for type ii resistance to fusarium head blight in wheat germplasm pi 672538. *Phytopathology* **2016**, *107*, 92-99.
21. Robinson, M.D.; McCarthy, D.J.; Smyth, G.K. Edger: A bioconductor package for differential expression analysis of digital gene expression data. *Bioinformatics* **2010**, *26*, 139-140.
22. Lovell, J.T.; Shakirov, E.V.; Schwartz, S.; Lowry, D.B.; Aspinwall, M.J.; Taylor, S.H.; Bonnette, J.; Palacio-Mejia, J.D.; Hawkes, C.V.; Fay, P.A. Promises and challenges of eco-physiological genomics in the field: Tests of drought responses in switchgrass. *Plant Physiol.* **2016**, *172*, 734-748.
23. Li, X.; Liu, T.; Chen, W.; Zhong, S.; Zhang, H.; Tang, Z.; Chang, Z.; Wang, L.; Zhang, M.; Li, L. Wheat wcbp1 encodes a putative copper-binding protein involved in stripe rust resistance and inhibition of leaf senescence. *BMC Plant Biol.* **2015**, *15*, 1.
24. Berger, S.; Sinha, A.K.; Roitsch, T. Plant physiology meets phytopathology: Plant primary metabolism and plant–pathogen interactions. *J. Exp. Bot.* **2007**, *58*, 4019-4026.
25. Rawat, N.; Pumphrey, M.O.; Liu, S.; Zhang, X.; Tiwari, V.K.; Ando, K.; Trick, H.N.; Bockus, W.W.; Akhunov, E.; Anderson, J.A. Wheat fhb1 encodes a chimeric lectin with agglutinin domains and a pore-forming toxin-like domain conferring resistance to fusarium head blight. *Nat. Genet.* **2016**.
26. Rustenholz, C.; Choulet, F.; Laugier, C.; Šafář, J.; Šimková, H.; Doležel, J.; Magni, F.; Scalabrin, S.; Cattonaro, F.; Vautrin, S. A 3,000-loci transcription map of chromosome 3b unravels the structural and functional features of gene islands in hexaploid wheat. *Plant Physiol.* **2011**, *157*, 1596-1608.
27. Sandhu, D.; Gill, K.S. Gene-containing regions of wheat and the other grass genomes. *Plant Physiol.* **2002**, *128*, 803-811.
28. Wicker, T.; Zimmermann, W.; Perovic, D.; Paterson, A.H.; Ganal, M.; Graner, A.; Stein, N. A detailed look at 7 million years of genome evolution in a 439 kb contiguous sequence at the barley hv‐eif4e locus: Recombination, rearrangements and repeats. *Plant J.* **2005**, *41*, 184-194.
29. Rustenholz, C.; Hedley, P.E.; Morris, J.; Choulet, F.; Feuillet, C.; Waugh, R.; Paux, E. Specific patterns of gene space organisation revealed in wheat by using the combination of barley and wheat genomic resources. *BMC Genomics* **2010**, *11*, 1.
30. Serrano, I.; Audran, C.; Rivas, S. Chloroplasts at work during plant innate immunity. *J. Exp. Bot.* **2016**, erw088.
31. Hill-Ambroz, K.; Webb, C.A.; Matthews, A.R.; Li, W.; Gill, B.S.; Fellers, J.P. Expression analysis and physical mapping of a cdna library of fusarium head blight infected wheat spikes. *Crop Sci.* **2006**, *46*, S-15-S-26.
32. Bauriegel, E.; Herppich, W.B. Hyperspectral and chlorophyll fluorescence imaging for early detection of plant diseases, with special reference to fusarium spec. Infections on wheat. *Agriculture* **2014**, *4*, 32-57.
33. Ajigboye, O.O.; Bousquet, L.; Murchie, E.H.; Ray, R.V. Chlorophyll fluorescence parameters allow the rapid detection and differentiation of plant responses in three different wheat pathosystems. *Funct. Plant Biol.* **2016**, *43*, 356-369.
34. Berridge, M.J.; Irvine, R.F. Inositol phosphates and cell signalling. *Nature* **1989**, *341*, 197-205.
35. Nourbakhsh, A.; Collakova, E.; Gillaspy, G.E. Characterization of the inositol monophosphatase gene family in arabidopsis. *Front. plant sci.* **2014**, *5*.
36. Chaouch, S.; Noctor, G. Myo‐inositol abolishes salicylic acid‐dependent cell death and pathogen defence responses triggered by peroxisomal hydrogen peroxide. *New Phytol.* **2010**, *188*, 711-718.
37. Coram, T.E.; Huang, X.; Zhan, G.; Settles, M.L.; Chen, X. Meta-analysis of transcripts associated with race-specific resistance to stripe rust in wheat demonstrates common induction of blue copper-binding protein, heat-stress transcription factor, pathogen-induced wir1a protein, and ent-kaurene synthase transcripts. *Funct. Integr. Genomics* **2010**, *10*, 383-392.
38. Asai, S.; Yoshioka, H. Nitric oxide as a partner of reactive oxygen species participates in disease resistance to necrotrophic pathogen botrytis cinerea in nicotiana benthamiana. *Mol. Plant-Microbe Interact.* **2009**, *22*, 619-629.
39. Orozco-Cárdenas, M.L.; Narváez-Vásquez, J.; Ryan, C.A. Hydrogen peroxide acts as a second messenger for the induction of defense genes in tomato plants in response to wounding, systemin, and methyl jasmonate. *Plant Cell* **2001**, *13*, 179-191.
40. Barna, B.; Fodor, J.; Harrach, B.; Pogány, M.; Király, Z. The janus face of reactive oxygen species in resistance and susceptibility of plants to necrotrophic and biotrophic pathogens. *Plant Physiol. Biochem.* **2012**, *59*, 37-43.
41. Caplan, J.L.; Mamillapalli, P.; Burch-Smith, T.M.; Czymmek, K.; Dinesh-Kumar, S. Chloroplastic protein nrip1 mediates innate immune receptor recognition of a viral effector. *Cell* **2008**, *132*, 449-462.
42. Belhaj, K.; Lin, B.; Mauch, F. The chloroplast protein rph1 plays a role in the immune response of arabidopsis to phytophthora brassicae. *Plant J.* **2009**, *58*, 287-298.
43. Gou, J.-Y.; Li, K.; Wu, K.; Wang, X.; Lin, H.; Cantu, D.; Uauy, C.; Dobon-Alonso, A.; Midorikawa, T.; Inoue, K. Wheat stripe rust resistance protein wks1 reduces the ability of the thylakoid-associated ascorbate peroxidase to detoxify reactive oxygen species. *Plant Cell* **2015**, *27*, 1755-1770.
44. Li, B.; Dewey, C.N. Rsem: Accurate transcript quantification from rna-seq data with or without a reference genome. *BMC Bioinformatics* **2011**, *12*, 1.
45. Mortazavi, A.; Williams, B.A.; McCue, K.; Schaeffer, L.; Wold, B. Mapping and quantifying mammalian transcriptomes by rna-seq. *Nat. Methods* **2008**, *5*, 621-628.
46. Anders, S.; Huber, W. Differential expression analysis for sequence count data. *Genome Biol.* **2010**, *11*, 1.
47. Liu, W.; Zhihui, W.; Yufeng ZHANG, D.G.; Yuzhou, X.; Weixia, C.; Haiying, Z.; Mingshan, Y.; Baoyun, L. Transcriptome analysis of wheat grain using rna-seq. *Front. Agr. Sci. Eng.* **2015**, *1*, 214-222.
48. Langmead, B.; Salzberg, S.L. Fast gapped-read alignment with bowtie 2. *Nat. Methods* **2012**, *9*, 357-359.
49. Kliebenstein, D. Quantitative genomics: Analyzing intraspecific variation using global gene expression polymorphisms or eqtls. *Plant Biol.* **2009**, *60*, 93.

**Table 1**. RNA-seq read information

| Tissue | Samples | Raw data | Clean reads | Mapped | Mapped % | Multiple | Multiple% | Unique | Unique% |
| --- | --- | --- | --- | --- | --- | --- | --- | --- | --- |
| spike | L661 spike at 0 HAI | 48619708 | 44064636 | 31413442 | 71.3% | 7948442 | 25.3% | 23472782 | 74.7% |
|  | L693 spike at 0 HAI | 54638926 | 50729857 | 37433618 | 73.8% | 15223441 | 40.7% | 22210177 | 59.3% |
|  | L661 spike at 24 HAI | 80401044 | 14261236 | 6799856 | 47.7% | 2277018 | 33.5% | 4522838 | 66.5% |
|  | L693 spike at 24 HAI | 56001617 | 37706168 | 24971426 | 66.2% | 7770208 | 31.1% | 17201218 | 68.9% |
|  | L661 spike at 72 HAI | 64589048 | 47444396 | 29577848 | 62.3% | 9209005 | 31.1% | 20368843 | 68.9% |
|  | L693 spike at 72 HAI | 73276052 | 52378107 | 33229807 | 63.4% | 9061381 | 27.3% | 24168426 | 72.7% |
| leaf | L661 leaf at 0 HAI | 74642980 | 40067567 | 28798631 | 71.9% | 9266948 | 32.2% | 19983610 | 69.4% |
|  | L693 leaf at 0 HAI | 53896907 | 37849318 | 18909880 | 50.0% | 8498338 | 44.9% | 10411542 | 55.1% |
|  | L661 leaf at 24 HAI | 52460707 | 45960283 | 35981574 | 78.3% | 20792164 | 57.8% | 15189410 | 42.2% |
|  | L693 leaf at 24 HAI | 54132118 | 45690464 | 32199399 | 70.5% | 20182030 | 62.7% | 12017369 | 37.3% |
|  | L661 leaf at 72 HAI | 50556369 | 26746381 | 18315598 | 68.5% | 9430647 | 51.5% | 6319347 | 34.5% |
|  | L693 leaf at 72 HAI | 49560263 | 26728939 | 18240700 | 68.2% | 10006376 | 54.9% | 8234324 | 45.1% |

**Table 2.** Pathways associated with the 13 genes in the gene island at region 181.40–181.54 Mb of wheat chromosome 3B

| **Pathways** | **Enzyme** | **Definition** | **Seq Nu.** | **Sequence** |
| --- | --- | --- | --- | --- |
| Oxidative phosphorylation | ec:1.10.2.2 - reductase | ubiquinol-cytochrome c reductase cytochrome b/c1 subunit | 7 | MSTRG.24508 |
|  | ec:1.6.99.3 - dehydrogenase | NADH dehydrogenase |  | MSTRG.24509, MSTRG.24524, MSTRG.24557, MSTRG.24558, MSTRG.24527, TRAES3BF007300290CFD_g |
|  | ec:1.9.3.1 - oxidase | cytochrome c oxidase cbb3-type subunit I |  | MSTRG.24509 |
|  | ec:1.6.5.3 - reductase (H^+^-translocating) | NADH:ubiquinone reductase (H+-translocating) |  | MSTRG.24509, MSTRG.24524, MSTRG.24557, MSTRG.24558, MSTRG.24527, TRAES3BF007300290CFD_g |
| Purine metabolism | ec:3.6.1.3 - adenylpyrophosphatase | adenosinetriphosphatase | 3 | MSTRG.24512, MSTRG.24554 |
|  | ec:3.6.1.15 - phosphatase | nucleoside-triphosphatase |  | MSTRG.24512, MSTRG.24554 |
|  | ec:2.7.7.6 - RNA polymerase | DNA-directed RNA polymerase subunit alpha |  | MSTRG.24544 |
| Glyoxylate and dicarboxylate metabolism | ec:4.1.1.39 - carboxylase |  | 2 | MSTRG.24551, MSTRG.24552 |
| Biosythesis of antibiotics | ec:4.1.1.39 - carboxylase |  | 2 | MSTRG.24551, MSTRG.24552 |
| Carbon fixation in photosynthetic organisms | ec:4.1.1.39 - carboxylase |  | 2 | MSTRG.24551, MSTRG.24552 |
| Thiamine metabolism | ec:3.6.1.15 - phosphatase |  | 2 | MSTRG.24512, MSTRG.24554 |
| Pyrimidine metabolism | ec:2.7.7.6 - RNA polymerase |  | 1 | MSTRG.24544 |
| Galactose metabolism | ec:3.2.1.26 - invertase |  | 1 | MSTRG.24512 |
| Phenylpropanoid biosynthesis | ec:1.11.1.7 - lactoperoxidase |  | 1 | MSTRG.24516 |
| Starch and sucrose metabolism | ec:3.2.1.26 - invertase |  | 1 | MSTRG.24512 |
|  | ec:3.2.1.48 - alpha-glucosidase |  | 1 | MSTRG.24512 |

Figure Legends

**Fig. 1.** Comparisons of L693 and L661 in terms of FHB severity and differentially expressed genes. **A** Photograph of spikes of L693 (left) and L661 (right) grown under greenhouse conditions 21 days after inoculation with *F. graminearum* in 2013. Disease symptoms are visible only at the inoculated L693 spikelet, but in the entire spike of L661. Bars, 1 cm. **B** Comparison of kernel health between the resistant line L693 (left) and susceptible line L661 (right) in 2013. Kernels of L693 were fully filled, while those of L661 were shriveled and pinkish. Bars, 1 cm. **C** Multiple comparison of the percentage of diseased spikelets (PDS) in 2013 and 2014. Error bars indicate the standard error of PDS; the letters above the error bars represent significant differences at a p-value of 0.01.

**Fig. 2.** Comparison of differentially expressed genes in L693 and L661 following FHB infection. Number of up-regulated genes (left, yellow circle), down-regulated genes (right, blue circle), and genes without differential expression (middle, purple circle) of L693 (resistant) compared to that of L661 (susceptible) at 0, 24, and 72 hours after inoculation (hai) in spike (**A**) and leaf (**B**).

**Fig. 3.** The reliability of RNA-seq data, as demonstrated by qRT-PCR and sample clustering. **A** Correlation between normalized mRNA-seq RPKM results and qRT-PCR expression values. Scatterplot shows the log_2_ fold change of RPKM and qRT-PCR expression values; a trend line is shown in red. **B** Sample clustering based on counts of Illumina reads mapped. Dendrogram represents the hierarchical clustering of samples as determined by Euclidean distance. The heat map shows a false-color representation of the sample correlation value.

**Fig. 4.** Log-fold change (logFC) against log-counts per million (logCPM) in different genotypes. **A-F** Differentially expressed genes with a false discovery rate (FDR) of less than 5% and a log-fold change of larger than 2 are highlighted in red. In each panel, red dots above the upper blue line (logFC > +2) represent up-regulated genes in L693 or downregulated genes in L661; red dots blow the lower blue line (logFC < -2) represent upregulated genes in L661 or downregulated genes in L693. The blue dots represent gene(s) with extreme values of –12 > logFC > 12, or with an average logCPM of > 10. Green symbols represent genes of interest in this study; rectangle for MSTRG.24516, circle for MSTRG.24551, triangle for MSTRG.24552.

**Fig. 5.** Venn diagram showing the number of differentially expressed genes shared between L693 and L661 at 0 h (red), 24 h (green), and 72 h (blue) after inoculation for the spike (**A**) and leaf (**B**). Numbers without parentheses represent differentially expressed genes across the whole genome; numbers in parentheses represent differentially expressed genes on wheat chromosome 3B.

**Fig. 6.** Distribution of genes on wheat chromosome 3B. **A** Eighty-six genes annotated by the Genetics Diversity Ecophysiology of Cereals (GDEC) group at the French National Institute of Agronomic Research (INRA) located on region 181.40–181.54 Mb of wheat chromosome 3B. **B** Fifty-three expressed genes located on region 181.40–181.54 of chromosome 3B in this study. Insets show enlargements of the boxed regions.

**Fig. 7.** Distribution of gene expression levels on chromosome 3B in all 12 samples. The x-axis represents the chromosome range 0–774.43 Mb. The y-axis represents the normalized log (RPKM+1) range 0–1. The region included in the dashed rectangle indicates the 181.40–181.54 Mb region. Distribution of gene expression level on chromosome 3B for six spike samples (**A**) and six leaf samples (**B**). Distribution of gene expression level on the gene island at region 181.40–181.54 Mb of chromosome 3B for six spike samples (**C**) and six leaf samples (**D**).

**Fig. 8.** A proposed working model of the roles of MSTRG.24516, MSTRG.24551, and MSTRG.24552 in the regulation of FHB resistance in wheat line L693.**A** In the early infection stage, FHB induces temporary susceptibility in local tissue; **B** this then leads to system-acquired resistance in distal tissue.
