# Supplementary material for: Transcriptome Analysis Identifies a 140 kb Region of Chromosome 3B Containing Genes Specific to Fusarium Head Blight Resistance in Wheat
Source: Int J Mol Sci. 2018 Mar 14;19(3):852. doi: 10.3390/ijms19030852 (PMC5877713; doi:10.3390/ijms19030852)
Supplement: Supplementary file 1 [file ijms-19-00852-s001.zip › Supplementary Material 1.pdf]

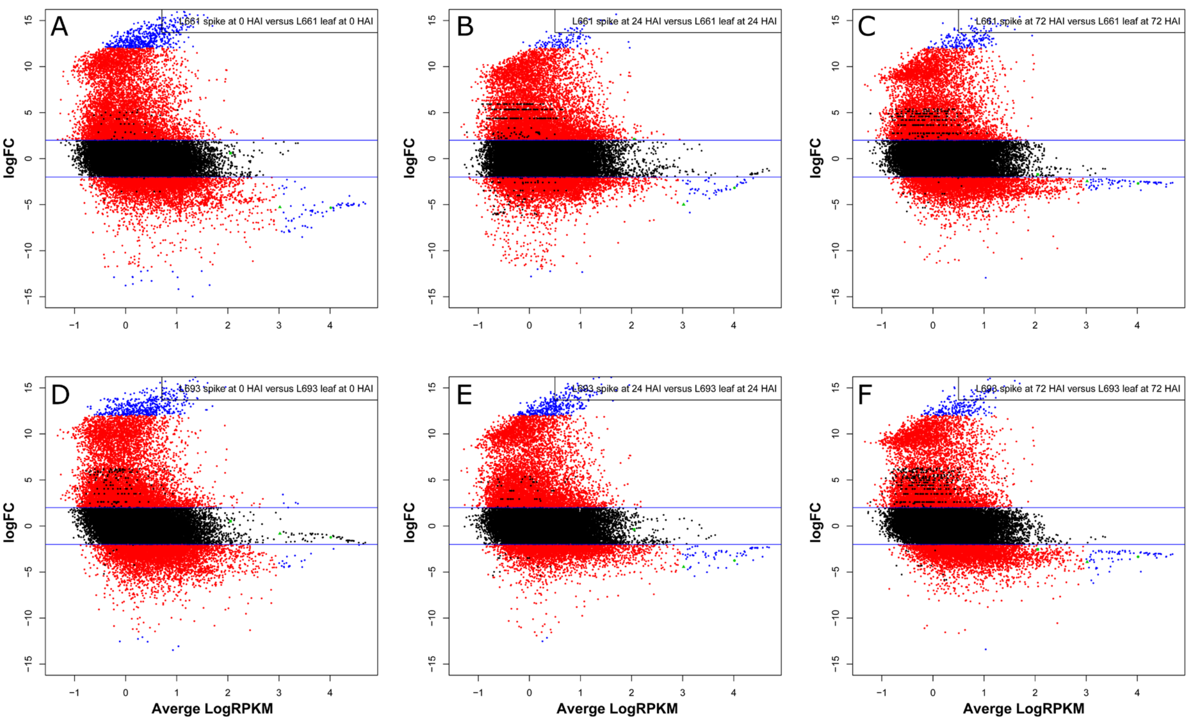

Figure S1. Log-fold change (logFC) against logRPKM in different tissues. Differentially expressed genes with an FDR of <5% and a log-fold change of >2 are highlighted in red. In each comparison, red dots below the blue line (logFC < 2) representing up regulated genes in leaf or down regulated genes in spike, in opposite, red dots above the blue line (logFC > 2) representing up regulated genes in spike or down regulated genes in leaf. The blue dots representing gene(s) with extreme value with -12 > logFC > 12 or have Average logRPKM more than 3. The green dots represent the interested gene in this study, rectangle for MSTRG.24516, circle for MSTRG.24551, triangle for MSTRG.24552.

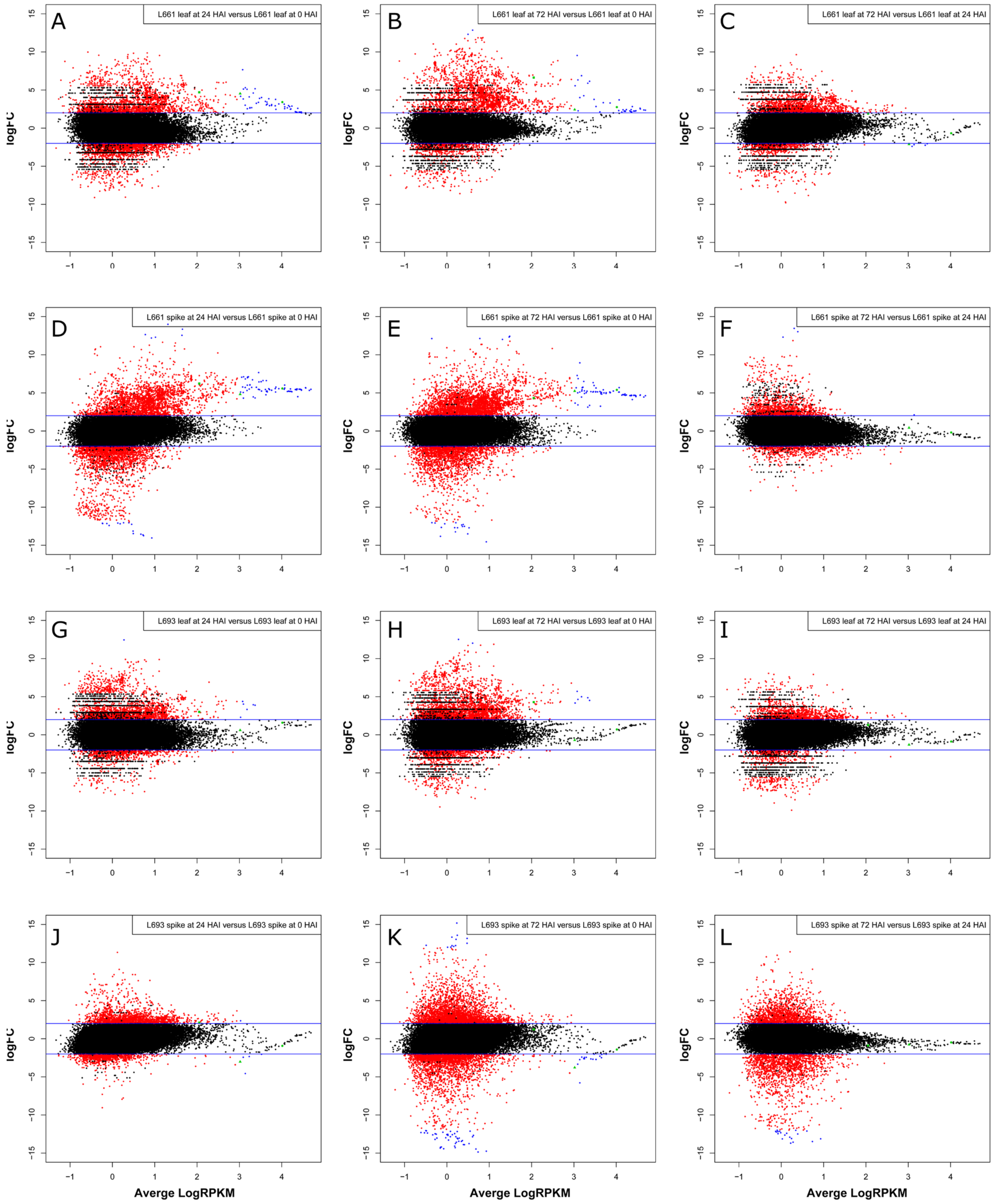

Figure S2. Log-fold change (logFC) against average logRPKM at different time points. Differentially expressed genes with an FDR of <5% and a log-fold change of >2 are highlighted in red. The blue dots representing gene(s) with extreme value with -12 > logFC > 12 or have Average logRPKM more than 3. The green dots represent the interested gene in this study, rectangle for MSTRG.24516, circle for MSTRG.24551, triangle for MSTRG.24552.

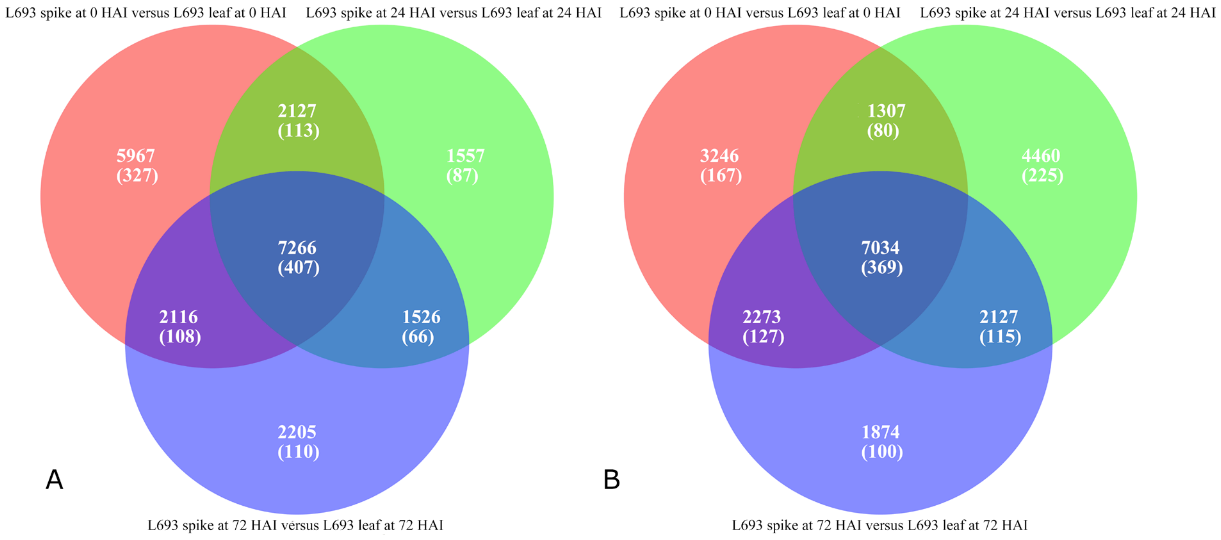

Figure S3. Venn diagram displaying the number of differentially expressed genes between the leaves and spikes of L693 (**A**) and L661 (**B**) plants at 0 h (red), 24h (green), and 72 h (blue). Numbers without parenthesis stand for different expressed genes over the whole genome, numbers with parenthesis stand for different expressed genes on wheat chromosome 3B.

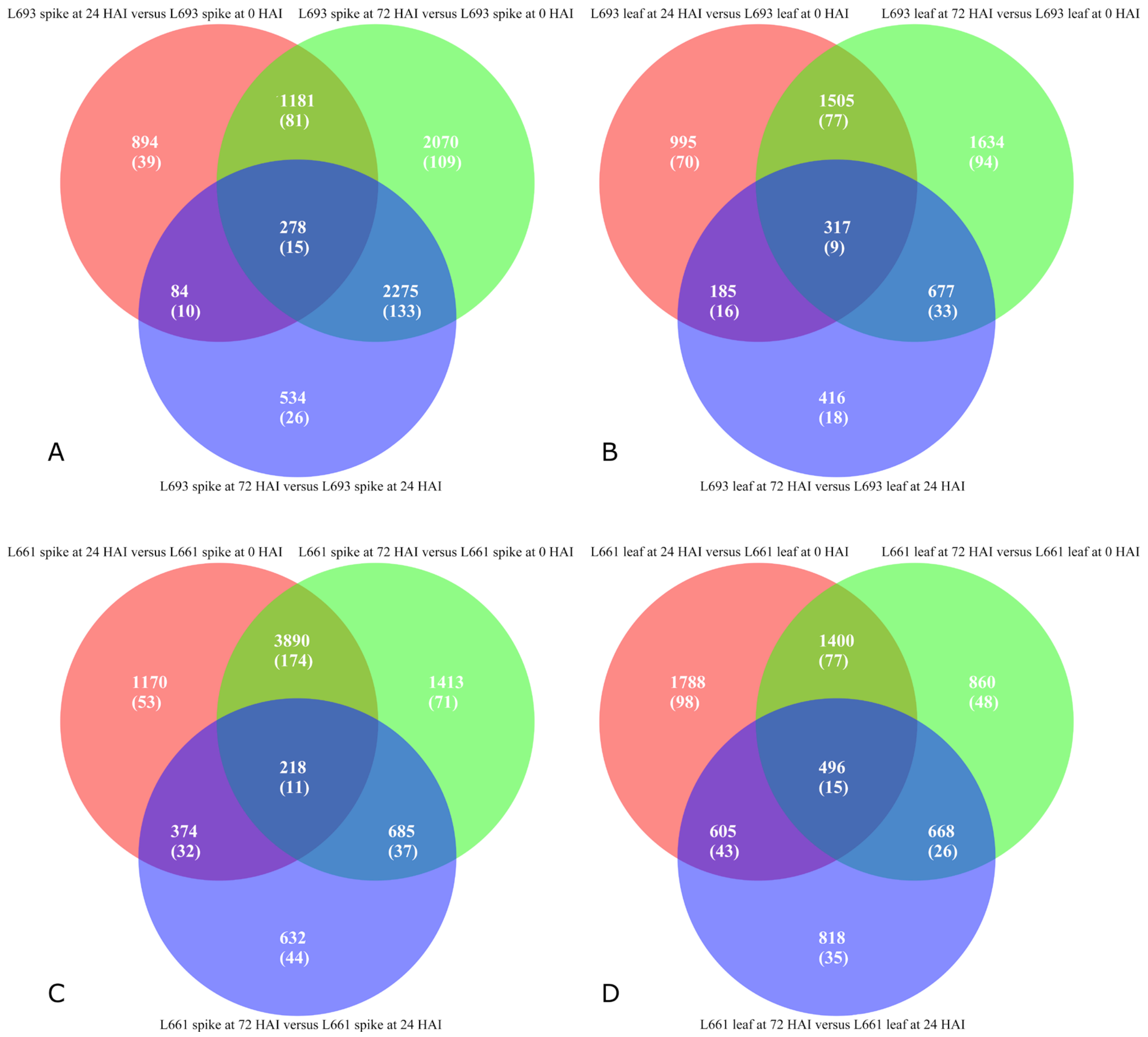

Figure S4. Venn diagram displaying the number of differentially expressed genes in the L693 spike (**A**), L693 leaf (**B**), L661 spike (**C**), and L661 leaf (**D**) at 0 h and 24 h (red), 0 h and 72 h (green), and 24 h and 7 2h (blue). Numbers without parenthesis stand for different expressed genes over the whole genome, numbers with parenthesis stand for different expressed genes on wheat chromosome 3B.

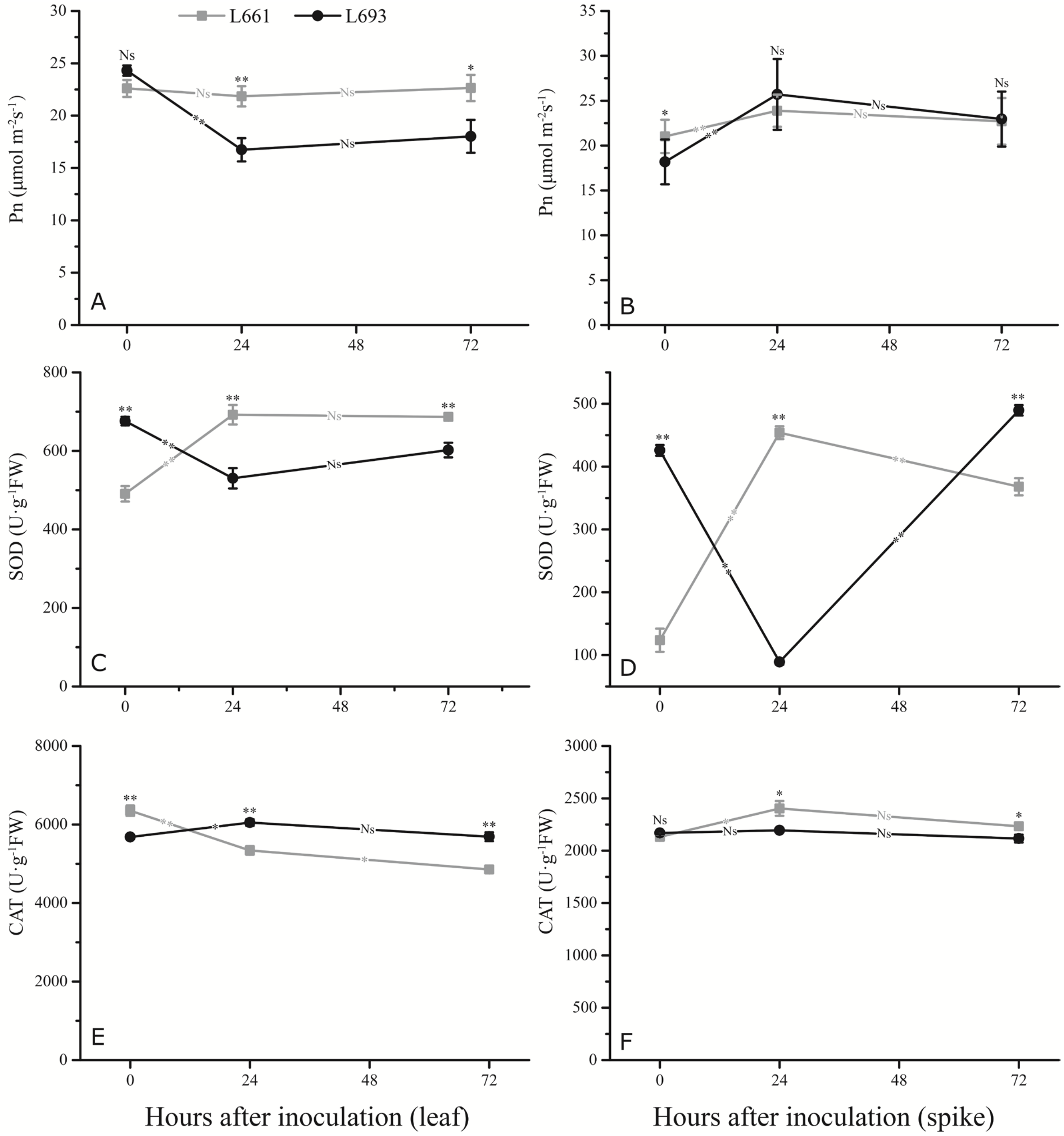

Figure S5. Changes in net photosynthetic rate (P_n) in the leaf (**A**) and spike (**B**), antioxidant enzyme activity (SOD) in the leaf (**C**) and spike (**D**), and catalase activity (CAT) in the leaf (**E**) and spike (**F**) following *Fusarium graminearum* inoculation.
