# Supplementary figures and images for: Transcriptome Analysis Identifies a 140 kb Region of Chromosome 3B Containing Genes Specific to Fusarium Head Blight Resistance in Wheat
Source: Int J Mol Sci. 2018 Mar 14;19(3):852. doi: 10.3390/ijms19030852 (PMC5877713; doi:10.3390/ijms19030852)

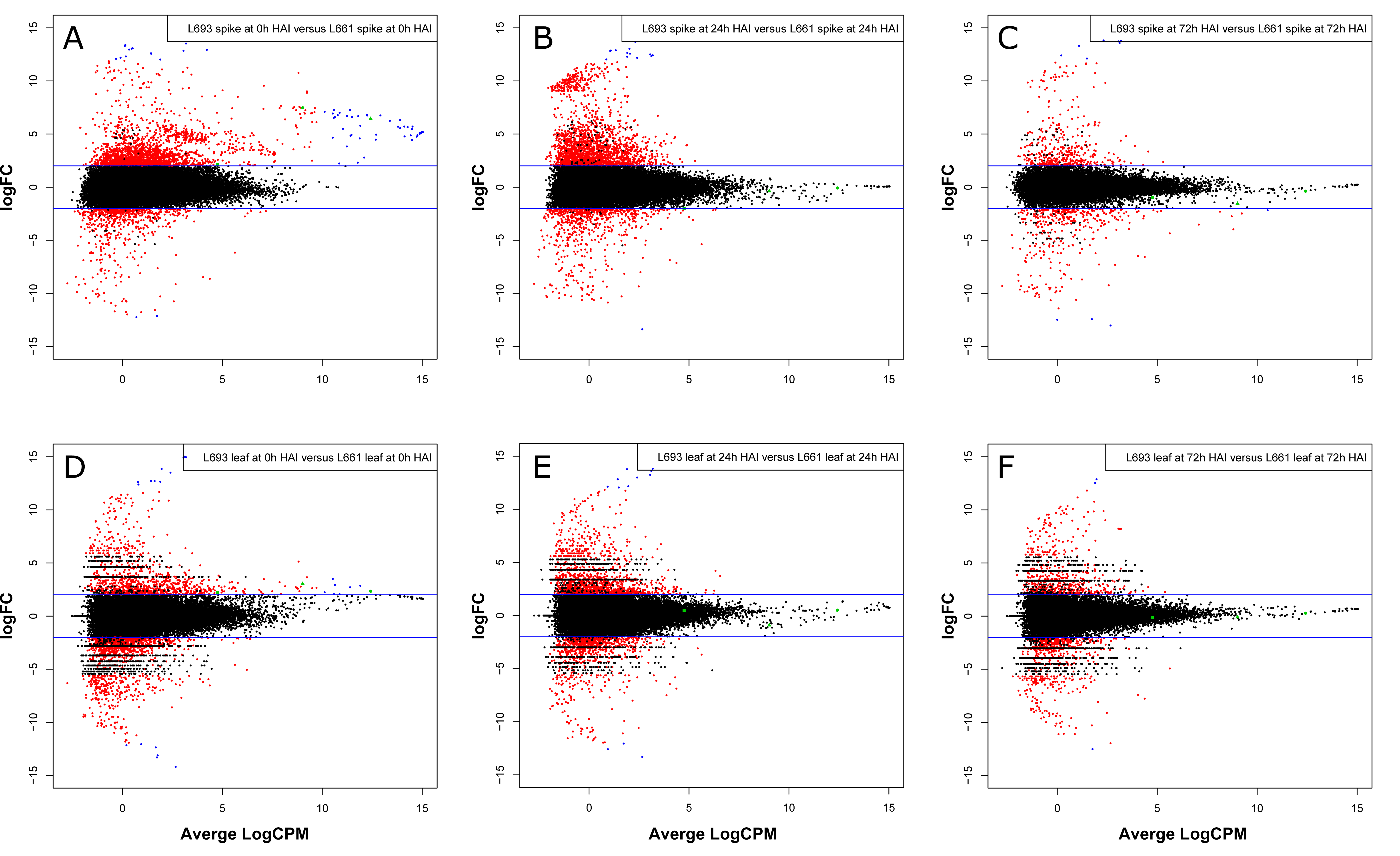

Supplement: Supplementary file 1 [file ijms-19-00852-s001.zip › Fig 4.tif]

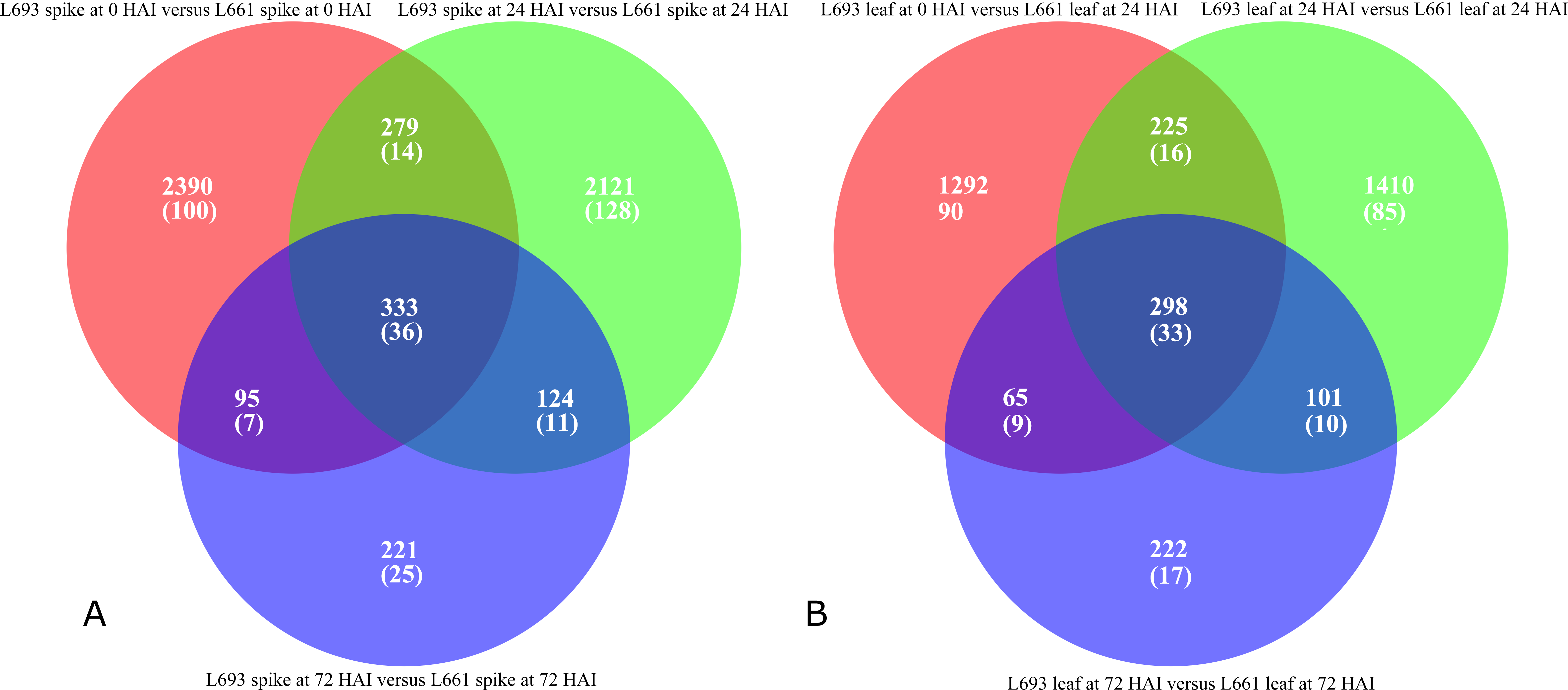

Supplement: Supplementary file 1 [file ijms-19-00852-s001.zip › Fig 5.tif]

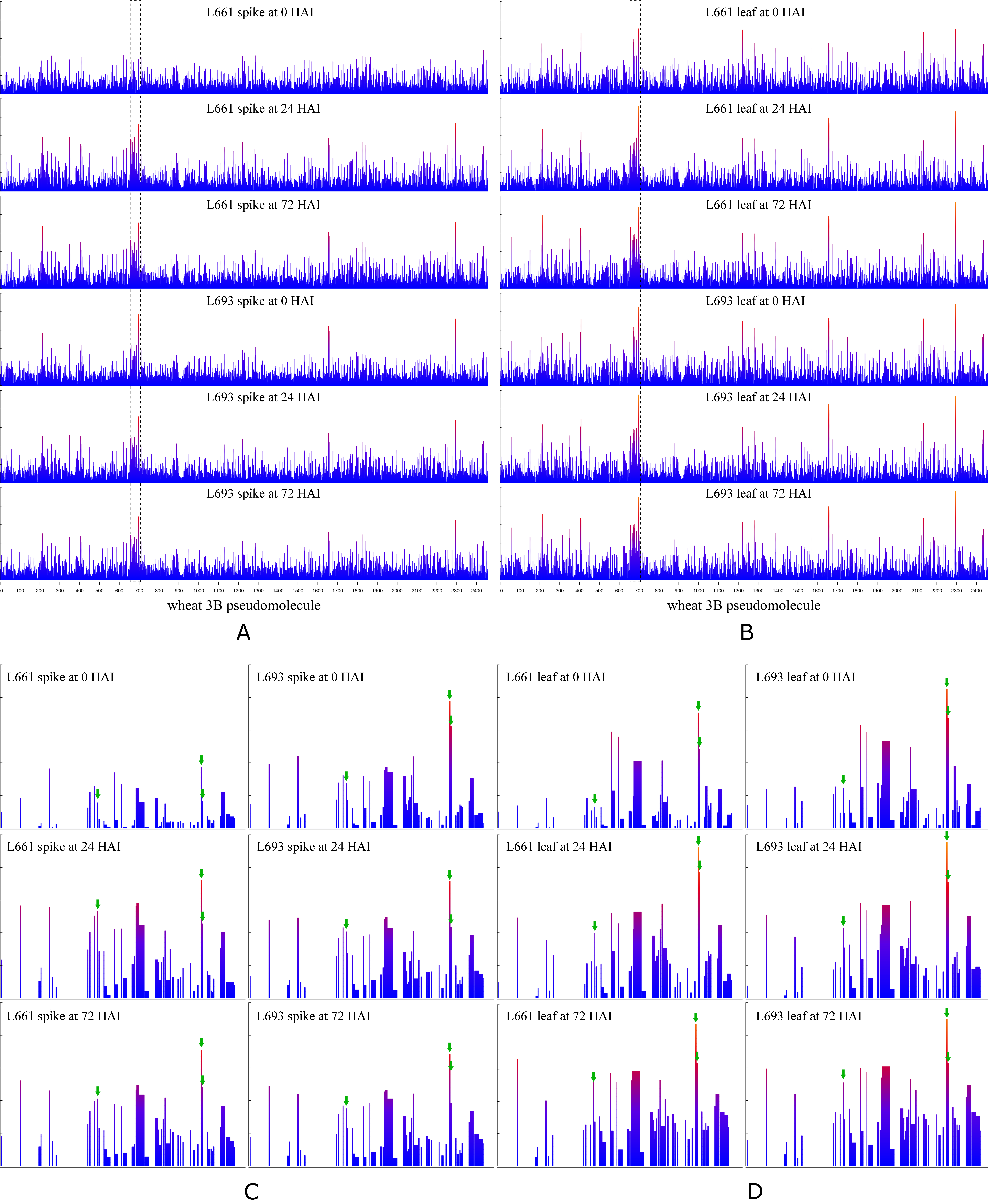

Supplement: Supplementary file 1 [file ijms-19-00852-s001.zip › Fig 7.tif]

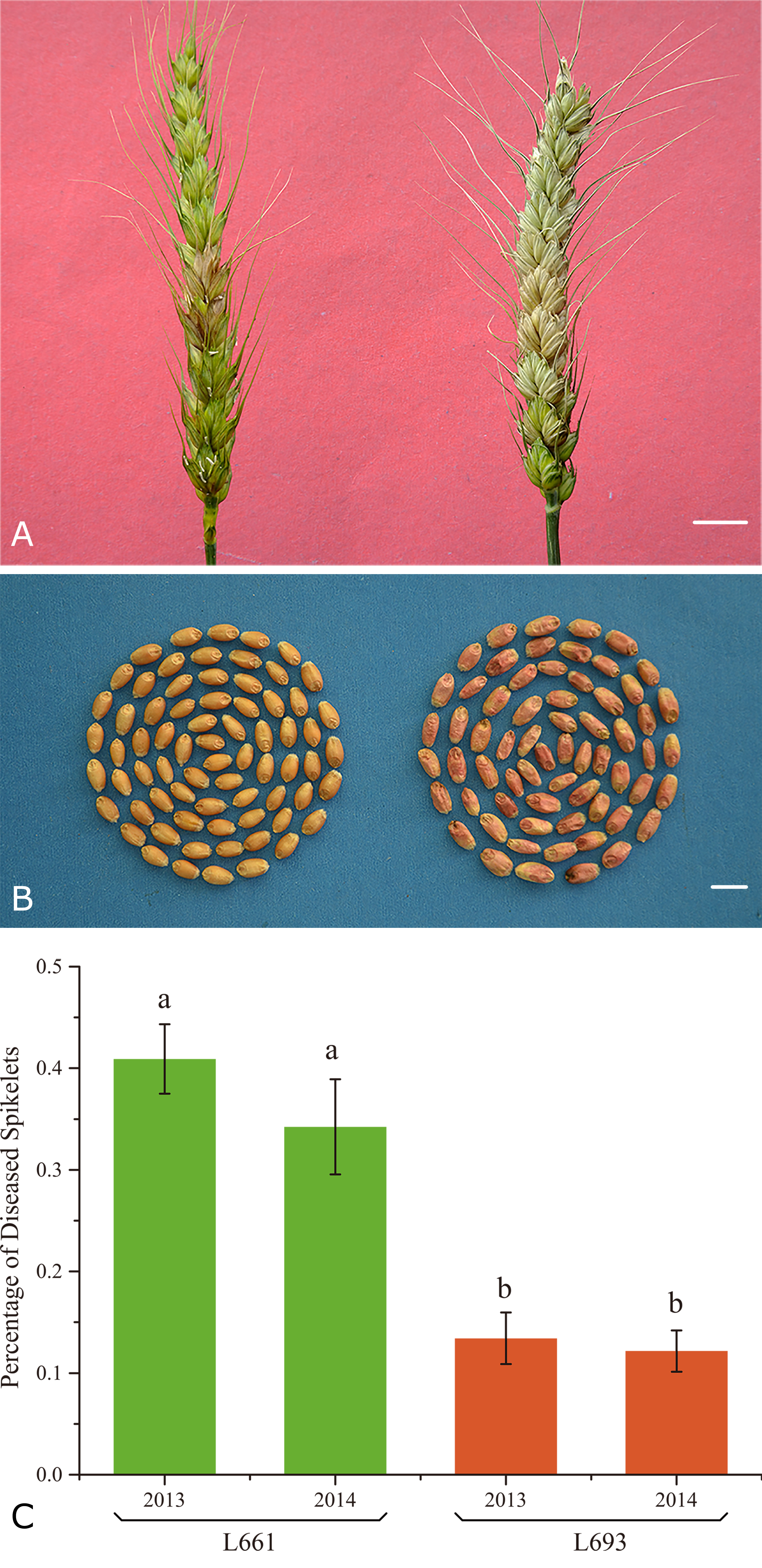

Supplement: Supplementary file 1 [file ijms-19-00852-s001.zip › Fig 1.tif]
